# Correction: Spatial regulation of Drosophila ovarian Follicle Stem Cell division rates and cell cycle transitions

**DOI:** 10.1371/journal.pgen.1011040

**Published:** 2023-11-13

**Authors:** David Melamed, Aaron Choi, Amy Reilein, Simon Tavaré, Daniel Kalderon

The published version of S1 Methods is incomplete. Please see the complete S1 Methods file below.

## Supporting information

S1 Methods The diagrams and text, divided into two major sections, “Live FUCCI Calculation Method” and “Sampling considerations for Live FUCCI Calculation Method”, provide an intuitive and mathematical explanation of how sdampling of multiple cells during live imaging with FUCCI reporters provides data suitable for calculating the absolute duration of G1 and G2 phases. (PDF)
