# Supplementary material for: Spatial regulation of Drosophila ovarian Follicle Stem Cell division rates and cell cycle transitions
Source: PLoS Genet. 2023 Sep 25;19(9):e1010965. doi: 10.1371/journal.pgen.1010965 (PMC10553835; doi:10.1371/journal.pgen.1010965)
Supplement: S1 Methods — (PDF) [file pgen.1010965.s001.pdf]

Sampling Considerations for Live FUCCI calculation method

1 A model for random sampling of cell cycle

In this section we describe a stochastic model for random sampling of a two-stage cell cycle. The cell cycle is represented as the circumference of a circle of length z divided into two phases, a first ("green") of length y, a second ("red") of length $z - y$. By symmetry, we may assume that the first phase starts at angle 0. We mimic the experiment by assuming a viewing window of length x whose starting angle is θ (reading in an anti-clockwise direction). We assume θ has a uniform distribution on $(0, 2\pi)$. See Figure 1. The data come from properties of the lengths of the red and green segments that are viewed in the interval of length x.

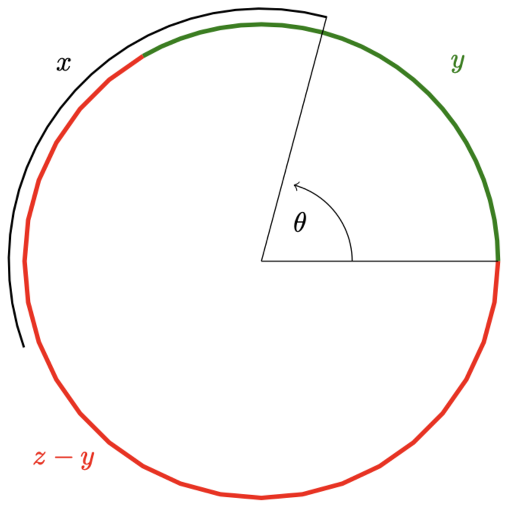

Figure 1: The green phase of length y shown on a circle of circumference z, the length of the cell cycle. The viewing window is of length x, and starts at an angle θ uniformly distributed over $(0, 2\pi)$.

1.1 Analysis when x, y, z are fixed

First we calculate the probability of several events related to this experiment, such as

- $\mathbb{P}(\mathrm{G})$, the probability that the entire observed region is green
- $\mathbb{P}(\mathrm{RG})$, the probability that the observation starts in the green region, and ends in the red region
- $\mathbb{P}(\mathrm{GR})$, the probability that the observation starts in the red region, and ends in the green region
- $\mathbb{P}(\mathrm{RGR})$, the probability that the observation starts in the red phase, covers the green region and ends in the green region
- $\mathbb{P}(\mathrm{R})$, the probability that the entire observed region is red
- $\mathbb{P}(\mathrm{G} \to \mathrm{R})$, the probability that an observation has green followed by red

and we also calculate $\mathbb{E}(G)$, the expected length of the observed green region.

There are two cases to consider: When $x < y$, elementary probabilistic arguments establish that

$$\begin{aligned}\mathbb{P}(\mathrm{G}) &= (y-x)/z, \quad \mathbb{P}(\mathrm{RG}) = x/z, \quad \mathbb{P}(\mathrm{RGR}) = 0, \quad \mathbb{P}(\mathrm{GR}) = x/z \\ \mathbb{P}(\mathrm{R}) &= (z-x-y)/z \text{ if } x+y<z; \quad 0 \text{ otherwise,}\end{aligned}$$

while if $y < x$, then

$$\begin{aligned}\mathbb{P}(\mathrm{G}) &= 0, \quad \mathbb{P}(\mathrm{RG}) = y/z, \quad \mathbb{P}(\mathrm{RGR}) = (x-y)/z, \quad \mathbb{P}(\mathrm{GR}) = y/z \\ \mathbb{P}(\mathrm{R}) &= (z-x-y)/z \text{ if } x+y<z; \quad 0 \text{ otherwise.}\end{aligned}$$

It follows that, in either case,

$$\mathbb{P}(\mathrm{G} \to \mathrm{R}) = \mathbb{P}(\mathrm{GR}) + \mathbb{P}(\mathrm{RGR}) = \frac{x}{z} \tag{1}$$

and

$$\mathbb{E}(G) = \frac{xy}{z}. \tag{2}$$

1.2 Estimating y

We will use equations (1) and (2) to estimate y from the data. To do this, begin by assuming that the values of y and z are fixed across n replicates of a given experimental condition, and write g_i for the length of the green segment observed in experiment i, and set $s_i = 1$ if experiment i results in an observation that has a green region followed by a red region, and 0 otherwise. From (2), we see that if x_i is the window length for experiment i, then $\mathbb{E}(g_i) = x_i y/z$, and so

$$\mathbb{E}\bar{g} := \mathbb{E}\left(\frac{1}{n}\sum_{i=1}^{n} g_i\right) = \left(\frac{1}{n}\sum_{i=1}^{n} x_i\right)\frac{y}{z} := \bar{x}\frac{y}{z}. \tag{3}$$

Similarly, from (1) we get

$$\mathbb{E}\bar{s} := \mathbb{E}\left(\frac{1}{n}\sum_{i=1}^{n} s_i\right) = \left(\frac{1}{n}\sum_{i=1}^{n} x_i\right)\frac{1}{z} = \bar{x}\frac{1}{z}. \tag{4}$$

The results in (3) and (4) suggest the ratio estimator $\hat{y}$ of y given by

$$\hat{y} = \frac{\bar{g}}{\bar{s}}, \tag{5}$$

since $y = \mathbb{E}\bar{g}/\mathbb{E}\bar{s}$.

Ratio estimators are biased, since (typically)

$$\mathbb{E}\hat{y} = \mathbb{E}\frac{\bar{g}}{\bar{s}} \neq \frac{\mathbb{E}\bar{g}}{\mathbb{E}\bar{s}} = y.$$

In order to estimate the bias in $\hat{y}$ we used the jackknife (see, for example, Choquet et al., 1999), which also provides an estimate of the variance of $\hat{y}$. The R package `bootstrap` was used for the analysis:

```
library(bootstrap)
# xdata is an n-row two-column matrix containing the values of
# (g_i,s_i) for given  experimental condition with $n$ observations
boottest <- function(xdata){
  n <- length(xdata[,1])
  thetaest <- mean(xdata[,1])/mean(xdata[,2])
  theta <- function(x,xdata){mean(xdata[x,1])/mean(xdata[x,2])}
  results <- jackknife(1:n,theta,xdata)
  return(c(thetaest,results))
```

The results are summarized in Table 1. Note that the corrected estimates are smaller than the original estimates, suggesting that the ratio estimator is biased upwards. The estimated standard error of the ratio estimator allows an assessment of the information carried about the average length of the green region in each experimental condition.

Dataset	Experiment	n	$\hat{y}$	bias	$\hat{y}_{\text{corr}}$	SE
1*	WT layer 1 G2	68	527.00	28.33	498.67	130.58
2*	WT layer 1 G1	24	81.05	1.62	79.43	19.47
3	WT layer 2 G2	59	1957.50	606.79	1350.71	1251.45
4	WT layer 2 G1	11	407.00	160.76	246.24	312.79
5*	CycE layer 1 G2	31	390.73	27.84	362.89	118.01
6*	CycE layer 1 G1	14	23.64	0.00	23.64	4.27
7	CycE layer 2 G2	21	1005.00	461.75	543.25	847.21
8	CycE layer 2 G1	9	180.20	36.93	143.27	118.53
9*	JAK layer 1 G2	44	277.33	11.55	265.78	65.85
10*	JAK layer 1 G1	45	204.95	7.38	197.57	46.535
11	JAK layer 2 G2	32	701.16	121.22	579.94	327.90
12	JAK layer 2 G1	19	550.75	145.42	405.33	325.95

Table 1: n denotes sample size. SE denotes standard error, corr denotes corrected estimate. Entries marked with * indicate plausible error estimate from SE.

1.3 Accounting for randomness in y and z

Next, we need to consider the effects of randomness in the values of y and z from experiment to experiment. We begin with a more detailed look at $\mathbb{E}\hat{y}$, by considering (y, z) as an observation from a random variable (Y, Z); we assume the replicates $(Y_i, Z_i), i = 1, 2, \ldots, n$ are independent across n replicates from the same experimental condition. Averaging over the distribution of (Y, Z),

we find from (1) and (2) that

$$\mathbb{E}(G) = x\,\mathbb{E}\left(\frac{Y}{Z}\right)$$

and

$$\mathbb{P}(\mathrm{G} \to \mathrm{R}) = x\,\mathbb{E}\left(\frac{1}{Z}\right),$$

so that

$$\mathbb{E}\bar{g} = \bar{x}\,\mathbb{E}\left(\frac{Y}{Z}\right), \quad \mathbb{E}\bar{s} = \bar{x}\,\mathbb{E}\left(\frac{1}{Z}\right). \tag{6}$$

and

$$\frac{\mathbb{E}\bar{g}}{\mathbb{E}\bar{s}} = \mathbb{E}\left(\frac{Y}{Z}\right) / \, \mathbb{E}\left(\frac{1}{Z}\right). \tag{7}$$

To relate the ratio on the right of (7) to the parameter $\mathbb{E}Y$ we are trying to estimate, we need to make some assumptions. For example, if the distribution of Y is closely concentrated around its mean (so Y is almost constant), we might approximate $\mathbb{E}(Y/Z)$ by $\mathbb{E}(Y)\mathbb{E}(1/Z)$, so that the ratio estimator in (5) is appropriate.

An alternative is to use an explicit model for (Y, Z), one of which we describe in the next section.

1.4 A model for (Y, Z)

One explicit model takes $Y = W_1, Z = W_1 + W_2$, where W_i has a gamma distribution with shape parameter $r_i > 0$ and scale parameter $1/\lambda > 0$, and W_1 and W_2 are independent. The density of W_i is

$$f_i(x) = \frac{\lambda^{r_i} x^{r_i - 1} e^{-\lambda x}}{\Gamma(r_i)}, \quad x > 0,$$

illustrated in Figure 2.

We have

$$\mathbb{E}Y = \frac{r_1}{\lambda}, \quad \mathrm{Var}Y = \frac{r_1}{\lambda^2}.$$

It follows that Z has a gamma distribution with parameters $r_1 + r_2$ and λ, and furthermore Y/Z and Z are independent. As a consequence,

$$\mathbb{E}Y = \mathbb{E}\left(\frac{Y}{Z}\,Z\right) = \mathbb{E}\left(\frac{Y}{Z}\right)\mathbb{E}Z,$$

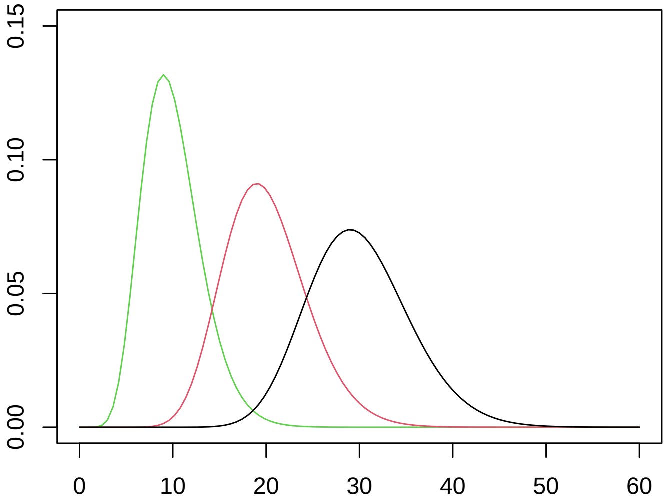

Figure 2: Illustrative densities of the green region W_1 (green line, shape =1, mean = 10), the red region W_2 (red line, shape = 1, mean = 20), $W_1 + W_2$ (black line, shape = 1, mean = 30).

so that

$$\mathbb{E}\left(\frac{Y}{Z}\right) = \frac{\mathbb{E}Y}{\mathbb{E}Z} = \frac{r_1}{r_1 + r_2}.$$

We also have

$$\mathbb{E}\left(\frac{1}{Z}\right) = \frac{\lambda}{r_1 + r_2 - 1}$$

The upshot of these results is that (7) becomes

$$\frac{\mathbb{E}\bar{g}}{\mathbb{E}\bar{s}} = \frac{r_1}{\lambda} \frac{r_1 + r_2 - 1}{r_1 + r_2} \approx \frac{r_1}{\lambda} = \mathbb{E}Y,$$

if for example $r_1 + r_2$ is large. It follows that the ratio estimator in (5) is still appropriate.

References

Choquet D, L'Ecuyer P, Léger C. Bootstrap confidence intervals for ratios of expectations. *ACM Transactions on Modeling and Computer Simulation*, **9**, 326–348, 1999.
