# Supplementary figures and images for: Spatial regulation of Drosophila ovarian Follicle Stem Cell division rates and cell cycle transitions
Source: PLoS Genet. 2023 Sep 25;19(9):e1010965. doi: 10.1371/journal.pgen.1010965 (PMC10553835; doi:10.1371/journal.pgen.1010965)

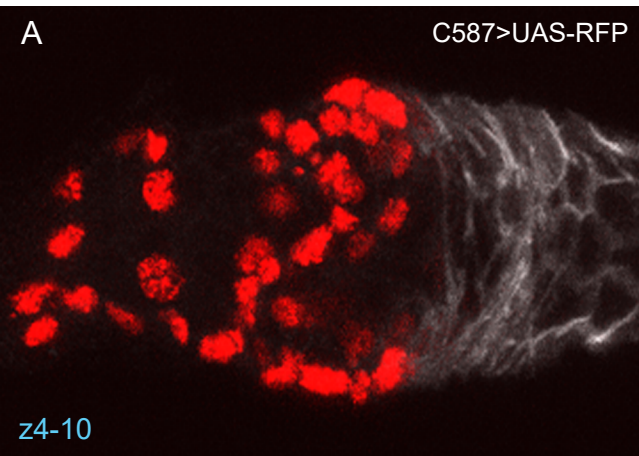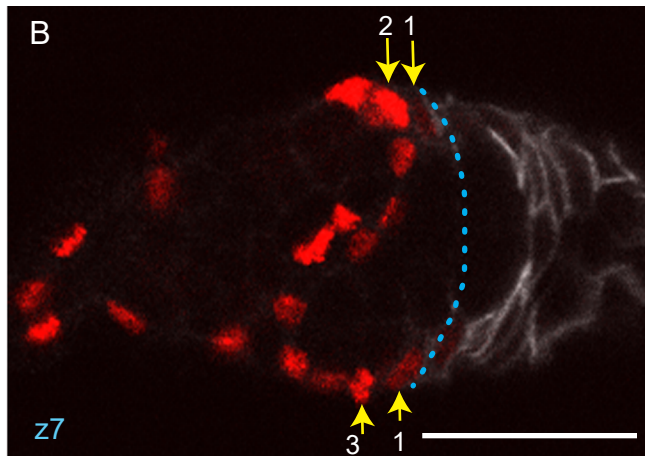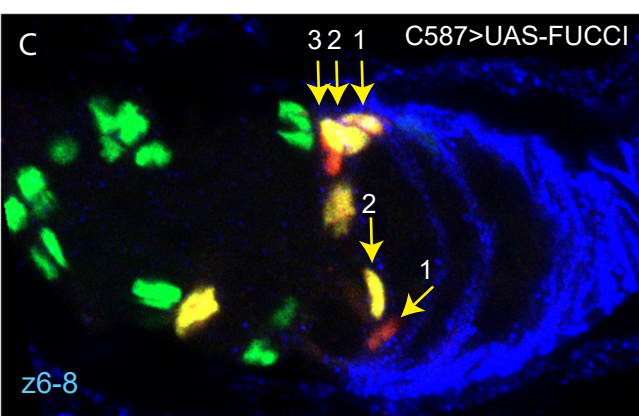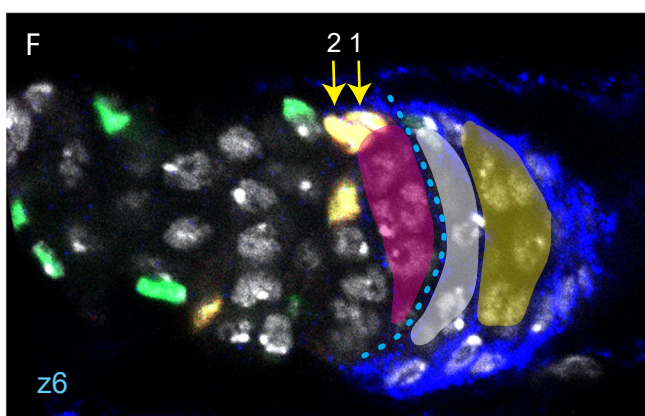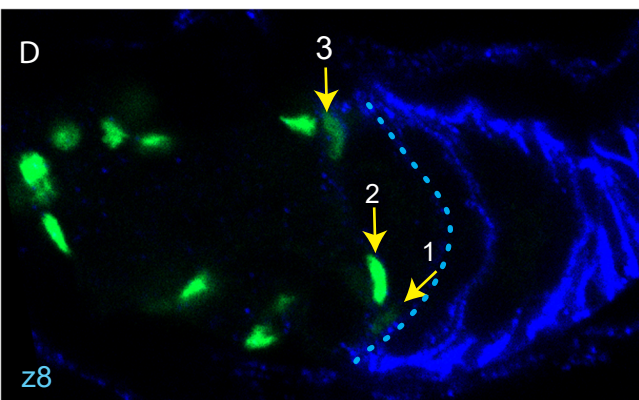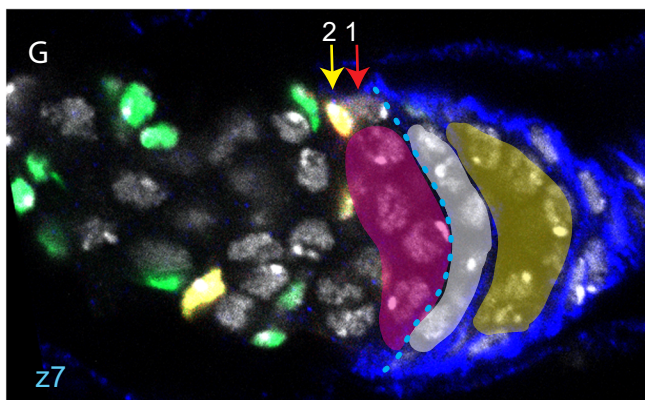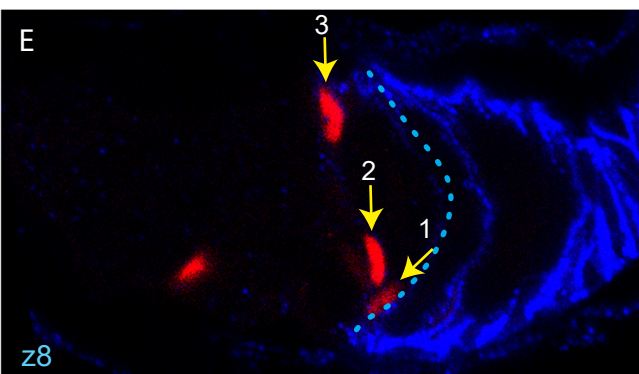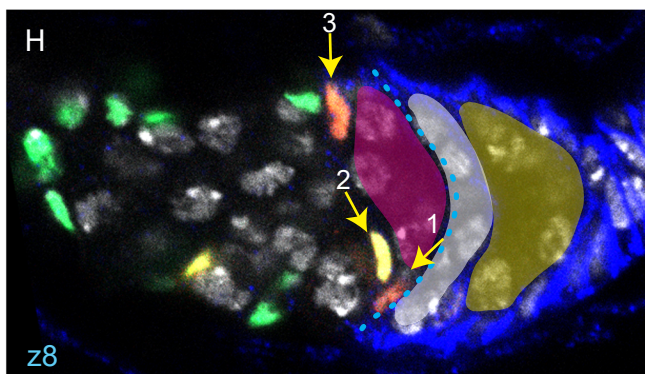

Supplement: S1 Fig — (A) Maximum projection of z sections spanning 15 μm of a C587>UAS-RFP germarium to show the C587-GAL4 expression pattern (red) relative to Fas3 staining (white). (B) Single z-section of the same germarium with the anterior border of strong Fas3 staining indicated with a blue dotted line, allowing designation of FSCs in layers (labeled as 1, 2 or 3). ECs and FSCs express RFP strongly, with weaker expression in Layer 1 FSCs and occasional weak expression in immediate FCs. (C-H) A C587>FUCCI germarium stained for DAPI (white) to show all nuclei. The anterior border of strong Fas3 (blue) expression is outlined by a blue dotted line in (D-H). FSCs in this germarium are in G2 (yellow arrows; GFP and RFP) or S phase (red arrow; no GFP or RFP). (C) Maximum projection of three z slices (z6-8) taken 2.5 μm apart, showing five FSCs. Three of these FSCs are shown in single-section images for z8 (D, E) and two others in z6 (F). Another FSC is visible only by examining z7 in isolation (G). (D) has only GFP and Fas3 channels, while (E) has only RFP and Fas3 channels, clarifying the presence of both GFP and RFP in the three indicated FSCs. (F-H) Individual, adjacent z sections showing germline cysts highlighted in different colors. Cysts are identified by clustered round germline cells which are larger than somatic cells. Identification of all germline nuclei allows even somatic nuclei with no GFP or RFP signal to be identified, including the layer 1 FSC in S-phase (red arrow) in (G). Most FSCs span two adjacent z sections: the Layer 2 cell in z6 (F) is also indicated in z7 (G). Note that layer 1 FSCs in (C-H) are immediately anterior to the anterior Fas3 border. Layer 2 FSCs are displaced roughly one cell body further anterior (left) and, in these examples, have a somatic cell nucleus between them and the Fas3 border. The single indicated layer 3 FSC is one cell diameter further anterior, neighboring a layer 2 FSC. Scale bar applies to all images; 20μm. (PDF) [file pgen.1010965.s002.pdf]

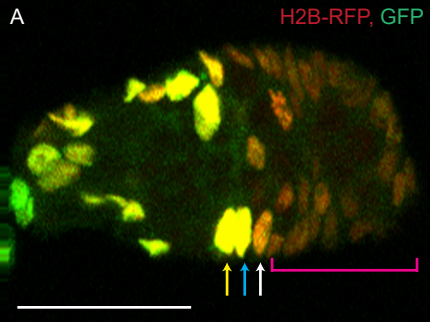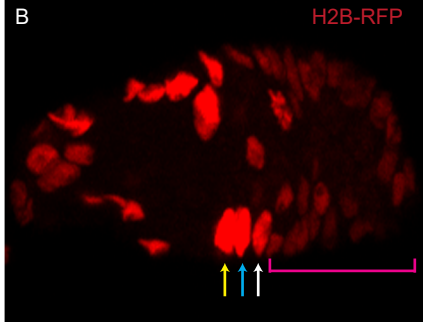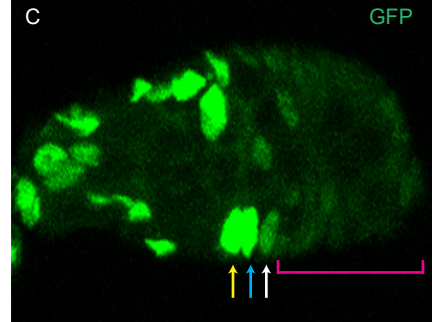

Supplement: S2 Fig — (A-C) Flies with UAS-H2B-RFP, UAS-GFP, actin-GAL4 and tub-tsGAL80 were kept at 29C for 4d and then fixed to show initial expression levels before any chase period at 18C. Both H2B-RFP (red) and GFP (green) showed strong expression in ECs and layer 2 and 3 FSCs (blue and yellow arrows), weaker expression in layer 1 FSCs (white arrows) and even weaker expression in FCs (pink bracket). Three consecutive middle z slices were combined for (A) RFP and GFP together, (B) RFP alone, and (C) GFP alone. Scale bar, 20μm. (PDF) [file pgen.1010965.s003.pdf]
