# Supplementary material for: Spatial regulation of Drosophila ovarian Follicle Stem Cell division rates and cell cycle transitions
Source: PLoS Genet. 2023 Sep 25;19(9):e1010965. doi: 10.1371/journal.pgen.1010965 (PMC10553835; doi:10.1371/journal.pgen.1010965)
Supplement: Attachment — Submitted filename: Review comments.pdf [file pgen.1010965.s012.pdf]

In the manuscript entitled ‘Spatial regulation of Drosophila ovarian Follicle Stem Cell division rates and cell cycle transitions’ by Melamed et al. use several methods including analysis of fixed and live imaging of FUCCI-labelled samples to determine the cell-cycle phase lengths in FSCs located in each of the three layers in the fly germarium. Additionally, the authors provide evidence for differential cell-cycle lengths for FSCs; these data are congruent with previous data from the same lab that FSCs in layer 1 divide 4X faster than the FSCs in layer 3. Additionally, the authors also describe how cell cycle lengths in these FSCs are regulated by gradients of various signaling pathways, and how manipulating these pathways affects later-specific cycling of FSCs, alluding to the differential sensitivity of FSCs to changes in these pathways.

In reviewing the manuscript first independently followed by in the light of previous review comments (and the authors’ response), I think two major concerns have been addressed satisfactorily: 1) the authors can identify FSCs in 3 layers faithfully, and 2) cells in S-phase can be identified faithfully.

My biggest hurdle in reviewing this manuscript, however, is that I (still) cannot understand the logic of how the cell-cycle lengths were determined despite extensive effort on the authors’ part to explain this: The logic and explanation of how these durations were calculated are described in response to previous reviewers, the text in the manuscript itself, and in supplemental data. After spending considerable time trying to read over theses sections over and over again and also to work this out independently (and failing to do so), it is impossible to say if the difficulty in grasping this concept is a reflection of how logic is presented or the logic itself. Either way, it is a struggle for readers, and the responsibility of making this clearer is on the authors. The readers are likely to undervalue the significance of the work if they don’t understand the material or worse, dismiss it altogether.

For example, in response to reviewers, the authors say:

Each sampling period begins at a random time relative to when a cell may have entered the phase we see at t=0. Imagine, for example, that G2 lasts 5h on average, that every imaging period is 2h and we capture cells distributed evenly at 30min intervals into G2 at (30, 90, 150, 210, 270 mins). [These are 60-minute intervals] Over 2h, those captured at 210 and 270 mins might (on average) exit G2 during imaging. We would have 5 cells imaged [what 5 cells? Are we assuming that there were 5 cells being tracked at t=0?] for 2h each (total 10h) [I don’t understand how time can be added like this. The imaging was done for a total of 2h] and found 2 that exit G2, so we deduce a G2 phase of 10/2= 5h [??]. Although the example uses regular intervals and uniform passage through G2, the logic applies equally when each is variable

In the manuscript, the authors say,
‘We reasoned that each cell in a specific phase (say, G2) was captured at an arbitrary

time within that phase at the start of imaging [this makes sense] so if we add together the total time spent by all cells of a specific type (say, layer 1 FSCs) in that phase over all germaria, **and divide by the number of transitions observed out of that phase** [Again, first I don't understand how time can be added like that (unless it is going to be divided by a denominator corresponding to the number of cells that stay in that phase i.e. time spent in a phase by a group of cells/number of cells in that phase at the end of the imaging period). Related to this, how does dividing that time by the number of cells that have left that phase (i.e. transition event) give you duration in the said phase?], we can infer the average phase duration.

Lastly, I appreciate the authors trying to come up with a mathematical model to explain the math. But that is even more difficult to understand.

Similarly, the math used to deduce S-phase duration is also difficult to understand.

Other points:

1) The titles of sections should be more descriptive of the topic described and be complete sentences. The most egregious examples is '**A G2/M barrier in posterior FSCs and a more prominent G1/S barrier further anterior'**
2) It makes more sense for Fig. 3 to be Fig. 2 and vice versa.
3) Abbreviations on lines 316 and 317 should be spelled out.
4) What do authors mean by 'after separating ovaries into individual ovarioles, 135 µL ovarioles were mixed with 15 µL Matrigel'?

My impression is that in general the authors have worked hard to make the text clearer; and certain sections are indeed clearer. However, there are still stumbling blocks that make it hard to understand and therefore appreciate the significance of the work presented.
