# Supplementary material for: Spatial regulation of Drosophila ovarian Follicle Stem Cell division rates and cell cycle transitions
Source: PLoS Genet. 2023 Sep 25;19(9):e1010965. doi: 10.1371/journal.pgen.1010965 (PMC10553835; doi:10.1371/journal.pgen.1010965)
Supplement: Attachment — Submitted filename: FUCCIRevResponseAug23.pdf [file pgen.1010965.s013.pdf]

Dear Editor,

All prior reservations of all four reviewers were addressed in earlier revisions with the exception of those expressed in the text pasted below from reviewer 4. The key issue is making plain the logic and veracity for calculating cell cycle phase periods from observations of fragments of cell cycles with live FUCCI reporter imaging (together with data from fixed imaging that shows the proportion of cells in a given location in each cell cycle phase). This question is thoroughly addressed in the response after the reviewer comments. Additionally, red text added to the reviewer comments addresses other minor points and clarifies specific issues regarding the main point that complement the extensive response to the main issue at the end.

The documents which have changed in the revised submission are:

1. Manuscript ("ManuscriptChanges" highlights changes made since last submission and is uploaded as a supplementary file. The current manuscript was combined with a pdf of Figures to make "CombinedManuscript&FiguresAug23", which is supplied as a supplementary file because the conversion process yields a pdf with very poor Figure resolution).

2. Revised Fig. 4 to update summary of cell cycle phase duration numbers and associated calculations.

3. Revised explanation of calculation method for determining cell cycle phase duration from live FUCCI imaging- "LiveFUCCICalculationMethodAug23" provided as a supplementary file.

4. An additional document to supplement the explanations above (in more formal mathematical language)- "Sampling Considerations for Live FUCCI Calculation Method" provided as a supplementary file.

5. Revised supplementary raw data spreadsheets- "Fig1,3,4,6DataRevModApr23ModJuly"

From Reviewer 4:
In the manuscript entitled 'Spatial regulation of Drosophila ovarian Follicle Stem Cell division rates and cell cycle transitions' by Melamed et al. use several methods including analysis of fixed and live imaging of FUCCI-labelled samples to determine the cell-cycle phase lengths in FSCs located in each of the three layers in the fly germarium. Additionally, the authors provide evidence for differential cell-cycle lengths for FSCs; these data are congruent with previous data from the same lab that FSCs in layer 1 divide 4X faster than the FSCs in layer 3.
Congruent in the sense that layer 1 divides the fastest, but quite different from earlier estimations using only EdU labeling in magnitude. The EdU index ratio between layers 1 and 2 was 1.3 but the newly-measured cell cycle time ratio is more than twice that, at 3.4 (because the length of S-phase is far from constant between the two layers). We made a couple of text adjustments to make this clear.
Additionally, the authors also describe how cell cycle lengths in these FSCs are regulated by gradients of various signaling pathways, and how manipulating these pathways affects later-specific cycling of FSCs, alluding to the differential sensitivity of FSCs to changes in these pathways.
In reviewing the manuscript first independently followed by in the light of previous review comments (and the authors' response), I think two major concerns have been addressed

satisfactorily: 1) the authors can identify FSCs in 3 layers faithfully, and 2) cells in S-phase can be identified faithfully.
Good- these are clearly major issues.
My biggest hurdle in reviewing this manuscript, however, is that I (still) cannot understand the logic of how the cell-cycle lengths were determined despite extensive effort on the authors' part to explain this: The logic and explanation of how these durations were calculated are described in response to previous reviewers, the text in the manuscript itself, and in supplemental data. After spending considerable time trying to read over theses sections over and over again and also to work this out independently (and failing to do so), it is impossible to say if the difficulty in grasping this concept is a reflection of how logic is presented or the logic itself. Either way, it is a struggle for readers, and the responsibility of making this clearer is on the authors. The readers are likely to undervalue the significance of the work if they don't understand the material or worse, dismiss it altogether.
This section is indeed important and we recognize the dual objective of (a) showing that the methods are valid and appropriate (to experts, including mathematical or statistical reasoning) and (b) explaining the concepts and methods in language that triggers understanding in as wide a group of readers as possible.
For example, in response to reviewers, the authors say:
Each sampling period begins at a random time relative to when a cell may have entered the phase we see at t=0. Imagine, for example, that G2 lasts 5h on average, that every imaging period is 2h and we capture cells distributed evenly at 30min intervals into G2 at (30, 90, 150, 210, 270 mins). [These are 60-minute intervals indeed- apologies for this trivial but confusing error (this is not in the manuscript)] Over 2h, those captured at 210 and 270 mins might (on average) exit G2 during imaging. We would have 5 cells imaged [what 5 cells? Are we assuming that there were 5 cells being tracked at t=0? Yes, and that they were 30, 90, 150, 210 and 270 mins into G2 at t=0] for 2h each (total 10h) [I don't understand how time can be added like this. The imaging was done for a total of 2h] and found 2 that exit G2, so we deduce a G2 phase of 10/2= 5h [??]. Although the example uses regular intervals and uniform passage through G2, the logic applies equally when each is variable
The material previously included as a mathematical explanation and now greatly expanded to include intuitive and statistical emphases is much more comprehensive than the explanation (in blue above) previously offered to supplement text changes, as a response to reviewers. I suggest ignoring the explanation above and concentrating instead on the comprehensive materials to be included in the manuscript.

In the manuscript, the authors say,
'We reasoned that each cell in a specific phase (say, G2) was captured at an arbitrary time within that phase at the start of imaging [this makes sense] so if we add together the total time spent by all cells of a specific type (say, layer 1 FSCs) in that phase over all germaria, **and divide by the number of transitions observed out of that phase** [Again, first I don't understand how time can be added like that (unless it is going to be divided by a denominator corresponding to the number of cells that stay in that phase (the denominator does indeed depend on how many cells stay in the phase because only those that exit are included in the denominator) i.e. time spent in a phase by a group of cells/number of cells in that phase at the end of the imaging period). Related to this, how does dividing that time by the number of cells that have left that phase (i.e. transition event) give you duration in the said phase?], we can infer the average phase duration.
The best non-mathematical answer to this question will be found in the first "Intuitive" section of the explanation in the supplementary file "LiveFUCCICalculationMethodAug23").

Lastly, I appreciate the authors trying to come up with a mathematical model to explain the math. But that is even more difficult to understand.
The explanations in this section have been slightly altered (and expanded) for easier understanding (and, more importantly, a new "Intuitive" section has been added). The central idea is that simple probabilities of the imaging period falling within a specific section of duration t within the whole cell cycle of duration T (probability = t/T) are first formulated as equations. This approach produces one equation for the expected time observed in the given phase over n cell samples, and another equation for the number of observed transitions out of the cell cycle captured in observation periods over n samples. These are exactly the two experimental parameters measured (total time in the phase over n cells, and number of exits from the phase over n cells). The desired underlying parameter (duration of the cell cycle phase) can be derived from these two equations and equals the ratio of the two measurements because all other parameters (time window, total cell cycle time) "cancel out".

Similarly, the math used to deduce S-phase duration is also difficult to understand.
Fixed imaging analysis allowed accurate measurement of the proportion of cells in each phase, for example for WT layer 1 FSCs. Hence, if we can work out, from live imaging, the absolute duration of any one phase, then we can work out the duration of each phase. We actually estimated from live imaging the duration of G1 and of G2 [also M] (more information than we need). We could work out S-phase length from either value and then compare. Instead, we chose to combine G1 plus G2 estimates. Thus, for example, imagine the measured duration of G1 + G2 (plus M, which is very short) is T mins and that 30% of the cell population (in layer 1) is in S-phase (so, 70% is in G1+G2+M), then the duration of S-phase is (30%/70%) multiplied by T. We believe that the estimates of the duration of G1 and of G2 are more or less equally good because the ratio of the two is very similar to the known relative frequency of G1 and G2 cells from fixed-sample FUCCI measurements. This is explained in the main text.

Other points:
1) The titles of sections should be more descriptive of the topic described and be complete sentences. The most egregious examples is "A G2/M barrier in posterior FSCs and a more prominent G1/S barrier further anterior"
Several section headings have been changed.
2) It makes more sense for Fig. 3 to be Fig. 2 and vice versa.
I find that it is more common and appropriate to show examples of images (raw data) first, before displaying the quantitative results from all such analyses graphically. Here, the graphs are very extensive, so that two Figures are required.
3) Abbreviations on lines 316 and 317 should be spelled out.
PI3K abbreviation for Phospho-Inositide 3' Kinase was already given in line 67. Here, now added again together with 92E suffix.
4) What do authors mean by 'after separating ovaries into individual ovarioles, 135 µL ovarioles were mixed with 15 µL Matrigel'?
Now changed to "After separating ovaries into individual ovarioles in imaging medium, 135 µL medium containing ovarioles was mixed with 15 µL Matrigel (Corning),…."

My impression is that in general the authors have worked hard to make the text clearer; and certain sections are indeed clearer. However, there are still stumbling blocks that make it hard to understand and therefore appreciate the significance of the work presented.

Manuscript changes in response to the request to clarify how cell cycle phases are calculated from live imaging data.

We have made multiple responses to explain further how the durations of cell cycle phases are estimated from experimental data. These are divided into three sections, according to the preferred depth of engagement of a reader. Since it cannot be anticipated what will be of interest to different readers, these are set out at length in supplementary materials (referenced and summarized in changes to the main text). The two Supplementary Files are entitled "LiveFUCCICalculationMethodAug23" (corresponding to sections 1 and 2 below) and "Sampling Considerations for Live FUCCI Calculation Method" (corresponding to section 3 below).

1. Intuitive, broad explanation.

This lays out the overall conceptual thinking and is likely the type of explanation the reviewer was requesting (at least as a starting point). It has been tested locally. Three readers who did not initially really understand the method were quite clear after reading this (another was also clear but already understood from earlier explanations).

2. "Mathematical" explanation based on probability and even sampling

For me, the intuitive explanation needs to be checked by a mathematical explanation. This was already done in the last submission but it is now expanded in a few ways: (i) additional explanation, (ii) showing validity for viewing time less than or greater than the duration of the cell cycle phase being explored, (iii) careful statement of assumptions and exploration of diverse situations. Previously, the mathematical derivation was considered only for the situation where each cell being monitored had the same cell cycle characteristics. A section is now added that considers heterogeneity. If either the total cell cycle time or the duration of a specific phase is constant, the equations are unaltered. If both are variable, then the equations are different and the method will only give an approximate value for average duration of cell cycle phases (more on this below). The analysis we perform is on very tightly defined cell populations- FSCs in a precise A/P location. To the best of our knowledge, the FSCs in a single layer are equivalent (because of radial symmetry) and in identical environments (the only known heterogeneity is in the A/P dimension). Thus, all cells for which we are trying to derive cycling parameters are likely to behave similarly on the basis of known drivers of behavior. This cannot, of course, exclude stochastic or other unknown variations.

The mathematical generalities derived above are then checked by considering a number of illustrative examples with specific numbers. These examples confirm that the method gives the correct average for the duration of a cell cycle phase when all cells in the population have identical cell cycles or have either just a shared total cell cycle time or a shared duration of the phase being investigated. As predicted, when both cell cycle phase duration and total cell cycle duration differ extensively within the population, the estimation of the average for the phase duration is no longer exactly correct. The magnitude of the error is explored with different sets of examples (with large variations, plausibly greater than encountered for FSCs). These examples show that the error is in a predictable direction (an underestimate because faster cycling cells are over-sampled) and

moderate (6-11% in the examples). We also consider the impact of some cells not cycling at all and find a similar potential impact (less than 10%) for the specific scenarios considered.

Thus, we provide full reasoning and numerical examples to show that the method should and does give a correct answer for average duration of a cell cycle phase under a certain set of conditions (assumptions). These assumptions are basically "even sampling" (see next section) and near-uniform cell behaviors. We also explored how fairly extensive heterogeneity of cell cycling impacts the derived estimates and find that errors would likely be no more than about 10% for plausible guesses of the maximal extent of heterogeneity in our samples and would systematically be underestimates.

This "mathematical" section was sent to a professional mathematician (social acquaintance) and approved (with small revisions and the additional comments that mathematicians would use different nomenclature). I also entered into extensive consultation with an expert on mathematical modeling of biological behaviors, including stem cells. The same equations presented in this section were independently derived by Dr. Simon Tavare (using different language in the derivation), after explaining the experimental data. We chose to retain the original language in this supplementary file, expecting it would be more accessible to those who are familiar with basic probability reasoning but without specialist training in mathematics or statistics.

3. Statistical exploration

Both the probability calculations and the worked examples (and diagrams) in the above section assume "even sampling"- essentially that the start of imaging for multiple cells will be spread evenly over all possible times in the cell cycle. This would be true for an infinite number of samples and would be more likely the greater the number of samples. To understand better how reliable the method may be with limited numbers of samples, the problem was addressed with statistical methods. This effort was led by Dr. Simon Tavare (and he has therefore been added as an author). We collaborated previously with Dr. Tavare regarding modeling FSC behavior and the impact on FSC competition of altered division rates. Dr. Tavare specializes in mathematics and statistics and has provided that expertise to many published studies of cell dynamics, often involving modeling, to explore behaviors of other stem cells and of cells within cancers. We had several meetings and exchanges over a period of several weeks to be sure of full explanation of conditions. To ensure that this treatment is suitable for expert analysis by statisticians it was prepared by Dr. Tavare as a separate supplemental document. My understanding is that the statistical treatment is not routine, with some important conceptual challenges and a choice of possible approaches to improve the statistical estimates and estimate errors. The choice of methods presented performed better than an earlier selection, but other options are possible. The "improvements in estimates" by using the chosen statistical method are all in one direction (reductions) and, for the most important data (layer 1 wild-type FSCs), moderate in magnitude (less than 10%). We provide these estimates in the supplementary document but do not adjust our estimates in the main text because (i) the original estimates are derived in a very simple way that can be used generally, (ii) the "improvement in estimates" suggested by the statistical analysis are not well-justified for smaller samples, and (iii) this sampling error (using layer 1 Wild-type FSCs as an example) leads to an over-estimate (likely less than 10%), while the sampling error if there is heterogeneity leads to an under-estimate (likely

less than 10%). Overall, the straightforward simple method we use is widely applicable and likely to give a good estimate for sufficient samples.
The statistical approach has the advantage of also yielding an error estimate (included in the document). The estimated standard error for both G1 and G2 duration for layer 1 FSCs is between 20-25% of the calculated mean.

Minor adjustments to raw data from live FUCCI imaging.

Finally, because of the extensive scrutiny of the live imaging data and their further treatment, inter alia by additional statistical methods, we also checked through the original videos and our tabulation of data derived from them. This led to a few adjustments, as described below.
We show these changes (with red and blue highlighting) in the revised supplementary data spreadsheet (tabs related to Fig. 4). After these are scrutinized by reviewers, the font can be changed back to black.
(i) We added some more cells (red font in spreadsheets that show the changes)- these were mainly (a) cells that were originally RFP-only (S-phase) and (b) derivatives of mitoses observed during imaging (only in UAS-CycE and UAS-Hop experiments).
(ii) We had previously used the time at which a cell has a different state (for example, a previously G1 cell last seen at 40 mins that has now lost GFP signaling S-phase entry when viewed at, say, 60 mins was scored as being in G1 until 60 mins) to calculate the time in the earlier cell cycle phase. We revised this approach to subtract half of the imaging interval (for example, 10mins for an imaging interval of 20 mins to estimate that the transition took place at 50 mins) because we do not know when the phase change occurred within the interval between successive images (from 40-60 mins). These changes are in blue font; note that there are no changes to the time if a cell entered and exited a phase within the same movie, which was common for G1, because the same increment of uncertainty would be added for the start of the phase and subtracted for the end of the phase. All of these changes had only a small impact on final calculated values (G1 and G2 reductions for excess CycE were substantially the biggest changes).

Main text alterations regarding live FUCCI imaging:

Alterations in the main text of the Results (live imaging section) direct readers to the appropriate supplementary documents and outline the overall considerations (additions to Figure legends refer to the raw data in supplementary data spreadsheets). Small adjustments to citation of all calculated cell cycle phase lengths (paragraph above) were made in the text and on Fig. 4. An additional paragraph in the Discussion re-iterates the key issues associated with calculation of cell cycle phase lengths and argues for the general value (limitations and advantages) of this approach to measuring cell cycle phases. I find it surprising that this approach appears not to have been used extensively in the past. Hence, it seems appropriate to summarize the pros and cons in a prominent location.

We believe we have addressed the final concerns of the reviewer extremely thoroughly. We have explained the method for calculating cell cycle phase duration at three different levels to cater to different readers, and shown through theory and examples that the method we use is valid, albeit subject to statistical considerations of potential errors that depend on the depth of sampling and potential heterogeneity within each cell population studied as a single group.

In addition to the cited changes, we made some small alterations to the text, mostly related to live FUCCI imaging results, to improve clarity and highlight the most important points.
