# Supplementary material for: Spatial regulation of Drosophila ovarian Follicle Stem Cell division rates and cell cycle transitions
Source: PLoS Genet. 2023 Sep 25;19(9):e1010965. doi: 10.1371/journal.pgen.1010965 (PMC10553835; doi:10.1371/journal.pgen.1010965)
Supplement: Attachment — Submitted filename: FUCCIReviewResponse.docx [file pgen.1010965.s010.docx]

Response to review for PLoS Biology

Two major issues were cited for the decision not to accept the manuscript at PLoS Biology:

1. The manuscript “does not fundamentally change our understanding of the mechanisms governing stem cell cycle to the degree that would be required of a study in PLOS Biology.” This editorial judgment derives from a comment from reviewer 3, “In short, the experiments are well performed, the data are carefully quantified, analyzed, and interpreted, but the overall take away seems more like a refinement of the accuracy of previous observations rather than a fundamental new understanding of how the system works.”

On the other hand, reviewer 1 points out that live imaging with FUCCI cell cycle markers is an exciting approach and that “there is some exciting science here”. Reviewer 2 comments are enthusiastic about the overall impact of this study.

Clearly, each view has merit and the judgment regarding impact is subjective.

My own opinion is more clear-cut. There is almost no direct measurement of cell cycling rates in any adult stem cell paradigm. The live FUCCI data from this manuscript therefore leads the way and is the primary reason why this paper is important as an example to be emulated for the whole stem cell field.

Second, the FSC paradigm is outstanding and hugely under-appreciated, providing an excellent venue for understanding (a) the roles of multiple known signals in regulating stem cell cycling and (b) how cell cycle rates relate to the overall dynamics of a (complex) stem cell population.

This paper provides several answers to those important questions, including (i) the cell cycle transitions targeted by different pathways, (ii) the contribution of graded JAK-STAT signaling to spatial heterogeneity of cycling (and inference that there must be other spatially restricted inputs), and (iii) how this spatial heterogeneity contributes to the dynamic equilibrium among members of the stem cell community in distinct A/P locations.

Spelling out these principles and reaching definitive conclusions makes the presentation of the FSC paradigm in this paper exceptionally interesting for the general reader.

I believe that the expertise of reviewers may have led some or all to discount or omit to cite this important aspect of our work. The other contributing factor for differences to my own evaluation of impact is likely that we did not spell out a clear enough picture of the background, what exactly was known previously and what was established in this paper. Accordingly, several changes have been made (highlighted in red), especially to the Summary and Introduction sections.

I believe there is really no question that this paper, the specific paradigm and the fundamental questions being asked about adult stem cells are of major interest to many and should be found in “high-impact” journals. Importantly, the quality of the work presented and the conclusions have been approved or lauded by the reviewers (specific points are addressed below).

2. The manuscript is very long.

Reviewer 1 and the Editor join forces in suggesting the manuscript will be “intractable even to experts”. Reviewer 2 makes no mention of length or difficulty reading. Reviewer 3 also clearly understood the content thoroughly but said that the paper is “so exceedingly long that it likely will be intractable to a general audience, and maybe even to a Drosophila savvy audience”; reviewer 3 also makes some specific helpful suggestions.

We initially considered principally following the suggestions of reviewer 3 for specific cuts, but decided that we had to make more drastic changes. We chose to preserve all of the new approaches, using FUCCI and H2B-RFP dilution, but to preserve only the MARCM studies relevant to JAK-STAT action at G1/S and G2/M transitions, supporting the FUCCI data. Essentially, this means eliminating the extremely data-rich previous Figures 7-9. The matching section of text was perhaps particularly hard to assimilate towards the end of a long paper, partly because the approach was similar in all tests but results needed careful explanation and assessment. The subject of Figs. 7-9 includes some analysis of the potential mediators of different signals, including Yorkie and Cyclin E, and contributions of some additional regulators. The major new discoveries and virtues of our study, highlighted in item 1 above, are left intact.

The FUCCI studies presented here are entirely new and have the greatest impact. Consequently, they are explained very carefully (there are some tricky issues and we wish to be precise about methods and limitations). That contributes to length, but I believe this material is easily read (and examined in Figures) for any reader. It occupies the bulk of the Summary and Discussion. Our preference for careful, transparent and comprehensive reporting is what led us to select a journal that is not constrained by a tight word-limit, acknowledging there can be good reasons for papers to be of different lengths. With the substantial cuts described above, we believe the paper is of a reasonable length, engaging and fully transparent.

We have made other adjustments in response to other reviewer comments. I have annotated the full review comments below with responses (in blue).

Reviewer #1, Aaron N. Johnson (note, this reviewer has signed the review): 
The manuscript by Melamed et al aims to answer a fundamental question of stem cell biology: how does a single stem cell population produce independent lineages at different rates? In this study, Drosophila follicle stem cells (FSCs) were used to model stem cell behavior in vivo. FSCs give rise to both follicle cells (FCs) and escort cells (ECs) depending the on the anterior-posterior position of the FSC in the germarium. FSCs produce significantly more FCs than ECs, and previous studies that used EdU incorporation suggest posterior FSCs divide faster than anterior FSCs to generate numerically distinct cell types. To understand if there is in fact differential cell division between anterior and posterior FSCs, the authors developed an ex vivo culture system to live image FUCCI labeled FSC cell division and progenitor migration. The live imaging studies show posterior FSCs do in fact cycle faster than anterior FSCs and provide the first kinetics of the process. The authors go on to explore the role of known FSC cell cycle regulators (JAK/Stat, Hh, P13K, Yorkie) in governing FSC cell cycle checkpoints using EdU to evaluate cell cycle progression. These studies provide a genetically tractable means to observe stem cell behavior in live tissues, which could have a broad impact on our understanding of how stem cells maintain homeostasis.

Live imaging of stem cells with the FUCCI cell cycle markers is an exciting approach that could provide a means to visualize stem cell activation, self-renewal, and migration in real time. Coupling the power of Drosophila genetics with this system could further clarify a number of unresolved questions related to stem cell behavior. However, the authors have not presented the data or written the manuscript in a manner that effectively communicates the impact of their discoveries.  The problems with the manuscript are simply too extensive to list. I suggest the authors work with a close colleague or collaborator to make major revisions that will align the manuscript with the current conventions in the field which include, but are not limited to, scope, manuscript organization, figure organization, statistical analysis and data presentation, and image presentation. I believe there is some exciting science here, but the manuscript is so "in the weeds" that I'm not sure what the essential arguments are and if the data supports or refutes the authors conclusions.

Responses regarding length have already been described. A response to other presentation issues raised here is incorporated in the first response to reviewer 2 below.
Reviewer #2: 
In this manuscript, the authors used follicle stem cells (FSCs) in drosophila ovary germarium, a classic model of adult stem cell, to study cell-cycle control and differentiation of stem cells. They used comprehensive genetic tools, beautiful imaging of cell cycle markers, and detailed quantification to extensively evaluate the effect of multiple niche signals. The data are clearly presented and quantified.

I would like to note simply that this reviewer found the quality of images, presentation and quantification detailed and clear. Other than the issue of length, which is addressed by our drastic revision of content, it is genuinely difficult to know which specific issues reviewer 1 found problematic in the presentation (I might guess and hope they were principally with regard to the material we have eliminated since reviewer 1 clearly lays out the first parts of our study).

Major comments:
1.	There are multiple signaling pathways explored in the manuscript. It would be helpful for readers get a whole picture of their findings if the authors make a summary diagram/cartoon at the end.

Excellent suggestion (even though we have now trimmed some of the information). We have added a summary diagram, Figure 7.

2.	The authors describe three layers of FSCs in WT. I wonder if in mutants, there could be more (or less) layers of FSCs?

This is an interesting question we hope to pursue carefully. It is not, however, trivial because FSC identity must be shown by functional lineage results and there are no satisfactory methods presently to target lineages in a single AP (layer) location. Nevertheless, functional FSCs must divide at a significant rate, so whether manipulations *likely* alter the anterior extent of the FSC domain can be surmised presently by looking at cycling. Those data are presented. Very likely therefore, for example, increased JAK-STAT leads to cells in EC territory behaving like FSCs (hence, our phrasing of such cells in the manuscript as being in EC territory, rather than calling them ECs). We don’t believe we can go further than that because we have no functional proof that the cells anterior to the FSC domain definitively function as FSCs.

3.	The authors emphasize spatial regulation of stem cell division due to spatial gradient of niche signals, but FSCs also move in anterior/posterior positions. How does the signals control such movement? Although this might be beyond the scope of the manuscript, it would be worth discussion.

This is a major question. We have addressed it in outline in a previous publication (Melamed et al.), showing that the magnitude of both Wnt and JAK-STAT pathways regulates AP preferences of FSCs. We are currently trying to look at the mechanisms involved. In the original version of this manuscript, we describe the effects of various genotypes on FSC location (and differentiation to ECs and FCs) in the MARCM studies. In the revised version we have eliminated most of those studies. I believe that the increased focus only on cell cycling means there is no reason to discuss cell location responses to signals here, beyond what is said in the introduction. However, this comment caused us to revise the summary and introduction considerably in order to provide a fuller background regarding signals and the regulation of differentiation (principally, lines 24-26, 35-37, 92-100 in red).

Minor problems: 
1.	The authors show spatial gradient of Wnt, JAK-STAT, Hh in Fig.1A. What about the spatial distribution of Hippo, PI3K and Integrin in germarium?

Another important question. Reporters of Yki activity (ex-lacZ, diap-lacZ) have previously been examined in the germarium. We found no clear pattern (Huang et al., 2017) but we are not convinced how good these reporters are in the germarium, so we prefer to leave the matter open. We have tried to use PI3K pathway reporters but so far without success of a clear signal (with controls of reduced and increased activity). The pattern of integrin staining has also been presented previously (O’Reilly, Lee, Simon, 2008, originally). It is clearly higher in FSCs and FC regions than ECs but a precise pattern, or any measure of activity, is not known. We have now removed the Hippo/Yki and integrin sections from the manuscript, as well as MARCM studies relevant to PI3K signaling. The spatial distribution of activities would certainly be good to know better but we cannot anticipate doing that in a short time and much of the relevant material is no longer present in the manuscript.

2.	Please quantify C587 reporter activity over the FSC domain. Would the graded knockdown or overexpress in the FSC domain, if any, affect result interpretation?

The pattern of C587 relative to FSC layers was carefully shown in ref (10) and we now cite that on line 460.

I believe the general question is whether any C587-driven genotype might alter the C587 expression pattern and hence, secondarily alter the pattern of genotype alteration. For most C587 experiments, the major consideration is whether sufficiently strong C587 expression persists in the FSC domain to potentially alter cell cycles, with the precise spatial pattern of any such changes being a minor issue.

For most genotypes we see FUCCI changes, so there must be adequate C587 activity.

For Wnt reduction, there are no great FUCCI changes. However, we have previously examined this genotype with a Wnt pathway reporter (Melamed et al., 2020) and found strong reduction- essentially background levels over the whole EC and FSC domain (now cited in lines 275-277).

In the same study, we measured and reported JAK-STAT pathway reporter activity to find it is roughly even across the FSC domain with C587>Hop. This is the one situation where the pattern of C587 expression is critical because it is used to flatten the JAK-STAT gradient. We previously reported the quantitative outcome for a JAK-STAT pathway reporter using exactly the same conditions (3d after shifting to 29C for GAL80 inactivation) used here for FUCCI measurements (and saw exactly the same effect on EdU incorporation as reported previously). We re-phrased this information for greater clarity (lines 463-465).

3.	Please include n number in figure legends.

We consider it impractical to cite those numbers in the legends or on the bars of graphs because of space constraints. We do supply spreadsheets with the raw numbers, together with the percentages displayed in bar graphs, as well as error and significance calculations, which of course utilize the n values.


Reviewer #3: 
Melamed et al., explore the important question of what mechanisms maintain a stem cell population in situation where stem cell proliferation is uncoupled from stem cell daughter differentiation.  In other words, how is homeostasis achieved to maintain a steady population of stem cells that give rise to multiple types of differentiated cells?  Their system is the follicle stem cell population in Drosophila ovaries.  They use a variety of fixed and live cell imaging to carefully measure the length of the cell cycle and thus the rate of proliferation in three different layers of FSCs.  The conclude that a gradation of cell cycle rates in the three FSC layers (posterior fastest, anterior slowest) likely maintains stem cell homeostasis in this tissue.

They also manipulate the activity of several different signaling pathways, most notably JAK/STAT which has previously been shown to modulate FSCs division, to ask which pathways control these rates and at want point in the cell cycle they work.  A main conclusion from this part of the work is that there is a second graded signal controlling FSC division rates that is not JAK/STAT, but we don't know the identity of this signal.  Also, it seems that the signaling pathways that impact FSC division rates do so at both the G1/S and G2/M transitions, which is interesting but not really a fundamentally new observation or concept. 

In short, the experiments are well performed, the data are carefully quantified, analyzed, and interpreted, but the overall take away seems more like a refinement of the accuracy of previous observations rather than a fundamental new understanding of how the system works.

As described earlier, the measurement of actual cell cycle rates is a first for any stem cell paradigm, the analysis of G1/S and G2/M restrictions, as well as how they respond to different signals, is a first for FSCs, and the use of this technology resolved a prior paradox that AP graded cycling appeared to remain in the absence of JAK-STAT but was absent when JAK-STAT signaling was spatially uniform when assayed by EdU incorporation. The new data clearly show there must be another major spatial signal and suggest it primarily affects the G1/S transition. We have made several changes to the Summary and Introduction to clarify exactly what was understood previously and hence what is new and what impact the new findings have.

Part of the motivation for these studies is that there has been a recent evaluation of FSC numbers, and so some of the old cell cycle regulation data in these cells might not be accurate.  Although a reasonable premise for further inquiry, the conclusions don't dramatically change from what we already knew, in part because the new approaches bring new caveats that also complicate interpretations.  This is not a criticism of the data, just that the new approaches don't fundamentally change our understanding of how signaling pathways modulation stem cell cycle regulation, or even how the process works in Drosophila FSCs.  That said, the live imaging of FSCs is new and powerful and a good addition to the field, even if the approach leads the authors to the same conclusions that layer 1 cells cycle faster and provide more FC whereas layer 3 cells cycle much slower and provide fewer ECs.

“Part of the motivation” is correct. That applies mostly to the MARCM studies of Figs. 7-9, which have now been eliminated. The entirely new virtues of the current revision are highlighted above, but I would also add that the quantitative measurement of cell cycle times show for the first time that FSC supply from layer 1 matches FC production. This has the important implication that there is no significant net flow of FSCs from anterior to posterior, allowing all FSCs to contribute similarly.

Although the detailed and thorough descriptions of the data are quite rigorous, the manuscript is so exceedingly long that it likely will be intractable to a general audience, and maybe even to a Drosophila savvy audience.  Some experiments don't extend the data in a meaningful way to justify their long description and even inclusion. For instance, the H2B dilution experiments are interesting but could be removed or at least included as supplemental data.  They do not provide conclusions different than FSC division rates in layer 1 > layer 2 > layer 3, and only add a few anecdotal instances of FSCs moving between layers.

Although “anecdotal”, this is the first direct evidence of FSCs moving in both directions. Prior lineage studies showed that two derivatives of a single FSC can end up in different layers but could not resolve if that was due to movement in just one, or both directions. We therefore chose to retain this material. It is part of the whole picture of a two-way dynamic equilibrium that is now solidly supported by comparing absolute measures of FSC divisions and new FCs produced per budding cycle. We believe it is important to provide direct evidence for as much of our understanding of FSC organization as possible.

Another example is the integrin experiments at the end of the Results: the data are somewhat equivocal and thus the conclusions uncertain. E.g. "it is presently unclear, but possible that integrin induction is important for JAK-STAT to induce cell cycling in EC territory".

This is now removed, along with far more.

Unfortunately the FUCCI system is nearly blind to positively scoring S phase cells because the cyclin B-RFP doesn't accumulate fast enough at the beginning of S phase.  The authors do a nice job of describing this.  However, this produces a potential problem when trying to score S phase using only the FUCCI system; i.e. do all quiescent cells express the E2F1-GFP reporter?  If not, then some of the colorless cells could be quiescent rather than in S phase.  The authors indicated that the number of colorless cells "was very similar to" the proportion of EdU positive cells, but they don't state how similar, nor do they explicitly state whether this assessment applies to all cells in the C587 positive region of the germarium or just the FSCs (I presume it's the former).  I suggest two things: 1) that the authors address the potential caveat of quiescence versus S phase being indistinguishable and whether they have data that can mitigate or dismiss it, and 2) whether it even
matters for their calculations.  For instance, if there were only a few quiescent cells called as S phase or vice versa it might not change their conclusions very much; i.e. what does "very similar to" mean exactly.  This all might seem a picky point, but the authors go to great lengths at being very careful and quantitative with their cell cycle measurements (which is applauded) and so perhaps this is suggestion is not out of scope here.

When EdU labeling was not used, the percentage of cells with no GFP (those assumed to be in S-phase) was 32.9% (81/246) for layer 1 and 24.0% (35/146) for layer 2. From a large number of MARCM experiments using control (normal) genotypes, the average EdU index was 33% for layer 1 and 20% for layer 2. (This explanation is now added to the text- lines 155-161). The very close match between these numbers suggests that all non-GFP cells are in S-phase. In other words, there is no mystery or uncertain category.

All of the results do indeed refer to the whole C587 territory (ECs and FSCs). In the EC territory, all DAPI-positive somatic cells have GFP (most have only GFP and are G1, a few also have RFP and are in G2). These ECs are non-cycling and might be termed quiescent (if they move into the FSC region they may cycle). If FSCs are “quiescent”, meaning they have a prolonged period of no progress through the cell cycle, I would expect on theoretical grounds that they would express GFP (and some RFP also), since there would have been no degradation of fluorescent protein for a long time and no barrier to expression of the fluorescent protein.

Altogether, I believe there is no strong theoretical reason to wonder whether some non-GFP cells are in a state of quiescence rather than S-phase, and the empirical data show that any potential inaccuracies in scoring S-phase cells as lacking GFP is very small. We have adjusted the text to avoid the ambiguity of “very similar”.

Is the wild type Dacapo expression pattern/level known in the C587 region, and might this provide an explanation for why the most anterior cells are refractory to Cyclin E overexpression?  Moreover, if Dap is normally very high in the EC's, this might explain why additional Dap didn't change the ability of Hop to drive them into S phase.

We have not examined Dap antibody staining. From scRNA-seq data, the levels of dap RNA are about 20% higher in ECs than FSCs but it is hard to be confident of the accuracy of those data. We also do not know by how much UAS-dap increases Dap levels. So, we cannot answer these interesting suggestions.

Editor’s summary:

While the reviewers appreciate the quality of the data presented and the use of refined tools, and reviewer 2 is more positive, reviewer 3 and our Academic Editor consider that the manuscript does not fundamentally change our understanding of the mechanisms governing stem cell cycle to the degree that would be required of a study in PLOS Biology. Our Academic Editor noted that the work does not provide sufficient insights into, for example, how changes in FUCCI profiles relate to FSC differentiation, and also raised issues with the conclusiveness the experimental design and data interpretations regarding cell cycle changes.

In this paradigm, we have shown in several earlier studies and state clearly in the introduction that FSC division and differentiation are uncoupled, with changes in division rate having little or no causative impact on differentiation. I am therefore puzzled why there should be any expectation that our work should extend to “how changes in FUCCI profiles relate to FSC differentiation”.

Second, what are the concerns about “experimental design and data interpretation”? I don’t see any specific comment about this from the reviewers- quite the opposite!

These both seems like unwarranted comments that cannot be defended as criticisms.

In earlier comments, I have outlined what is entirely new for the stem cell field and for FSCs in this paper. It is substantial.

The phrase “does not fundamentally change our understanding of the mechanisms… to a degree that would be required..” seems to me a boiler-plate rationalization, rather than a transparent yardstick that could be used in a balanced discussion.

**Summary of Changes**

Removal of Figs. 7-9 and associated text (p20-27 of Results, leaving p5-20) and almost one page of Discussion. Other small changes in the Summary, Introduction and Discussion (of course, methods, legends) to account for this change in content.

Alteration of text in response to specific comments, highlighted above- in red.

These changes particularly address the need for greater background regarding FSC differentiation and more precisely spelling out what was not previously known and why the missing information provided in this paper breaks new ground and contributes to important broad principles.

Addition of summary diagram, Fig. 7.

Inclusion (always available) of source data for Figures.
