# Supplementary material for: Spatial regulation of Drosophila ovarian Follicle Stem Cell division rates and cell cycle transitions
Source: PLoS Genet. 2023 Sep 25;19(9):e1010965. doi: 10.1371/journal.pgen.1010965 (PMC10553835; doi:10.1371/journal.pgen.1010965)
Supplement: Attachment — Submitted filename: PLoSGReviewMod1.docx [file pgen.1010965.s011.docx]

**Responses to Reviewers and Editor Comments**

Below, we reproduce Reviewer comments (black) and our responses (blue). At the end, we also address summary Editor comments.

Reviewer's Responses to Questions

**Comments to the Authors:
Please note here if the review is uploaded as an attachment.**

Reviewer #1: The study by Melamed et al investigates the important question of how stem cell proliferation rates are regulated, using the Drosophila follicle stem cells as a stem cell model. These stem cells exhibit population asymmetry and heterogeneous behaviors depending on their position in layers 1, 2 or 3. Although their differential proliferation rates have been previously documented by this group, how these different proliferation rates are governed and maintained is still unknown, and this paper addresses that question, using several methods including FUCCI imaging in fixed and live samples, dilution of a fluorescent protein, and clonal analysis. The paper was previously submitted to PLOS Biology, where it was rejected and sent with its prior reviews to PLOS Genetics.

We believe this summary greatly undervalues the impact of our work. The most important attribute of this paper (not mentioned in the above summary) is that absolute cell cycle times are measured for the first time for FSCs by using live imaging with FUCCI reporters. This produced the important result that layer 1 FSC division rates matched the previously measured rate of FC production. That result (i) provides further confirmation of the spatial organization, numbers and behavior of FSCs and (ii) suggests that the previously documented exchange between anterior and posterior FSCs is at equilibrium. The movement of FSCs in both directions is also shown directly in this paper for the first time. These insights go far beyond prior evidence of different division rates from EdU measurements, which are shown here to dramatically underestimate differences between FSCs in adjacent locations.

Unfortunately, the study has many serious methodological issues. Whether they can be fixed is not clear. Even without the problems in experimental design and missing data, the manuscript was so difficult to read that its greater significance would be lost on the reader. Thus, although my main concerns (see below) are more significant than writing style, clarity is also a major issue that needs addressing.

Each of the specific queries is answered below. Hopefully, it should be clear that the methods are very sound and, motivated by reviewer comments, now more fully explained. Clarity of presentation (including shortening some descriptions) has been improved further, but the specific comments of the reviewer on this subject appear to have been motivated by the content of our original submission to PLoS Biology, not the current manuscript (very large cuts were made). I suspect that the reviewer comments for the correct manuscript, let alone the newly-revised version would be much more favorable.

The first problem is that it seems to be difficult to recognize FSCs with precision. For example, in Fig. S1, the central green FSC, labeled as row 3, sits above and below cells that look identical to it and are at the same distance from the Fas3 border -- yet are not labeled as FSCs. How are such identifications made? There are very few examples of the actual image data that underlie this paper, and they give pause to whether the goals of this study can be met if FSCs are not identified by clonal methods. The authors must convince the readers that this is possible to do, especially important given the recent disagreements about the number and locations of the FSCs. In the methods they say that FSCs are identified by location, but that does not explain the cells above.

The issue of recognizing FSCs and their precise locations is discussed in the next response. Here we respond to the question of cells in Fig. S1 not labeled as FSCs.

All of the cells around the FSC region that are not labeled with arrows are germline cells. Germline cells will never label with UAS-FUCCI reagents driven by C587-GAL4 but it is essential to distinguish somatic and germline nuclei labeled by DAPI with no GFP or RFP signal. Germline nuclei are larger and more spherical. They are also clustered within specific geometries (lens-shape for stage 2b; rounded for stage 2a and 3). In the revised Fig. S1, each germline cyst is colored to clarify identity. The coloring was done by putting a dot at the center of each nucleus, going through all z-sections, checking for 16 nuclei in a cyst and coloring the inferred cyst. We have added to the Methods a description of how germline nuclei are distinguished from somatic nuclei.

If the question of identifying FSCs can be addressed, the next question is whether it is possible to accurately assign FSCs to layers 1,2,3. In Fig. S1, the uppermost three FSCs are assigned to layers 1 (top), 1 (middle), 2 (bottom). Yet the top and bottom FSCs seem to have equal claim to being in layers 1 and 2, although assigned differently, because each is partially blocked by the middle FSC and partially open to the Fas3 border. If there is no unambiguous way to assign them to layers, the analysis could still have value, as the somewhat arbitrary assignment still will reflect a trend (as a layer 1 FSC is not likely to be assigned to layer 3, and vice versa), but it will not have the kind of precision the authors use throughout the paper.

FSC designations are entirely by location. Previous studies in our lab, using exactly the same methods to define cell locations, provided the functional evidence that the designated cells serve as stem cells for FC production (hence, FSCs) (Reilein et al, 2017).

The reviewer raises a critical point, so we have taken several steps to clarify these issues.

It is very important that readers understand (i) we can identify the AP location of cells with excellent fidelity and (ii) we have demonstrated the functional properties of wild-type cells in these locations very extensively in prior publications. Indeed, those two activities have taken up the majority of all research time of three of the authors for the past several years, developing expertise that is without parallel.

(i) We have added precise explanations of labeled cell identities to the legends of Figure 1 and Figure S1. We also added the protocol we use to define FSC locations to the Methods.

All analyses depend on examining each of about 8 z-sections spanning the germarium. Hence, the process is best illustrated by the single z-sections shown in Fig. 1D and several parts of Fig. S1. In most other images (having illustrated the method and provided direct evidence of designated identities), we show the results of such analyses as a composite of a few z-sections in order to show a sufficient number of cells to show proportions in different cell-cycle phases.

When looking at a single z-section, starting from a central z-section, we first identify the border of strong Fas3 staining (fully described now in Methods). This is a convex line, bordering the posterior edge of a 2b cyst. In adjacent z-sections we follow this border (a convex surface that is more anterior near top or bottom z-sections). All cell locations (generally, only nuclei are marked) are then assigned relative to the Fas3 border in the same z-section. Nuclei adjacent to the Fas3 border are layer 1. Note that they will not all align in a Cartesian system on the image of one z-section because the Fas3 border is curved. This also applies to cells in different z-sections (which are sometimes present together in images showing multiple z-sections). Most layer 2 cells have a single somatic cell nucleus or part of a single nucleus between them and the Fas3 border in a single z-section. If there is no intervening nucleus, but the separation from the Fas3 border is clearly larger than for a layer 1 cell, it is also scored as layer 2 (not all conceivable layer 1 locations are occupied by a cell body in a given germarium). Layer 3 cells are one cell further from the Fas3 border (using analogous criteria). Each somatic cell nucleus is generally seen strongly in one z-section and partially (lower intensity, smaller) in just one adjacent z-section. It is therefore relatively easy to keep track of all somatic nuclei in layers 1-3 of a germarium without missing any cells or scoring any cell twice.

These procedures are extremely laborious and account for more than 80% of the time invested in an experiment and require considerable experience. There are, of course, limitations and challenges because germaria can have irregular shapes and the system is dynamic, so slightly different morphologies are captured at different times during the 12h budding cycle, but we are confident that our scoring is reproducible and accurate. Most important, it is consistent within this study and consistent with the methods used to define FSC locations by function. Many aspects of the data in this work also testify to the accuracy of our methods. If FSCs in different layers were regularly mis-scored in FUCCI-labeling experiments we would not end up with very different proportions of cells in each cell cycle phase in different layers (and the same proportions in S-phase as found in MARCM lineage studies). We also would not find a 3-4 fold difference in cycling rates between layers 1 and 2 from live imaging (and no evidence of any cell cycle transition for layer 3).

(ii) The functional evidence for FSCs residing principally in layers 1 and 2 is overwhelming. Since this was established in a paper published in 2017 and confirmed in additional ways in subsequent papers from this lab, we thought it would be sufficient just to summarize the conclusions and references for brevity. Since the reviewer suggests that readers may nevertheless remain skeptical, we have added a further couple of sentences of explanation and further references in the introduction (lines 84-102).

The next issues concern data reporting, and these are serious issues. A previous reviewer asked the authors to “include n number in figure legends.” The authors responded, “We consider it impractical to cite those numbers in the legends or on the bars of graphs because of space constraints. We do supply spreadsheets with the raw numbers, together with the percentages displayed in bar graphs, as well as error and significance calculations, which of course utilize the n values.” This response seems disingenuous, considering the length and wordiness of the manuscript; n values take only a few lines to report at most. But worse, the data are not reported in the supplement.

There are six graphs in the manuscript. For each, (Fig. 1B, Fig. 3A-D and Fig. 6D) we provide every n value in the supplementary data spreadsheets (contrary to the reviewer’s assertions). Please look at the bar graphs and consider (a) whether n numbers added to the graphs themselves is practical without creating a mess (it is not) and (b) whether it would be possible to describe the n values for each item in a Figure legend that would be readable.

We understand that most readers would prefer not to look at supplementary material, so we have added a general guideline to each legend of the range of numbers examined, as suggested by reviewer 2, referring also to the comprehensive supplementary data tables for precise details.

Additions to legends:

Fig. 1

SEM is shown from scoring 28 germaria (574 r1 ECS, 638 r2a ECs, 205 layer 3 FSCs, 323 layer 2 FSCs, 490 layer 1 FSCs).

Fig. 3

The number of cells scored ranged from 126 to 588 (average of 372) for layer 1 FSCs, 88 to 403 (average of 255) for layer 2 FSCs, 48 to 308 (average 165) for layer 3 FSCs, 190 to 755 (average 447) for r2a ECS, and 179 to 754 (average 426) for r1a ECs; precise numbers in supplementary data tables.

Fig. 6

All values derive from experiments where at least fifty ovarioles were scored. The total number of marked FSCs scored for an altered genotype ranged from 27-199 (average 101) for layer 1 FSCs, 28-139 (average 78) for layer 2 FSCs, and 24-169 (average 56) for layer 3 FSCs; precise values (for ECs also) are in supplementary data tables.

I can assure you that our response was not disingenuous and we do not take such accusations lightly.

I discovered this when trying to understand how the cell cycle time is calculated from the data. The authors state, “if we add together the total time spent by all cells of a specific type… and divided by the number of transitions observed out of that phase we could infer the average phase duration”. This is not at all clear to me. To try to understand, I looked for the actual data in the supplement, but it is not present. (Tangentially, the author’s summary of the weaknesses of the previous approach was also not clear: “The fraction of cells in M-phase or S-phase under different conditions will, however, only provide a measure of the relative cell cycle times if the length of that phase remains constant.” Please clarify, perhaps illustrating this statement with an example of how it could lead to errors.)

First, just the issue of transparency of data reporting. In the text, we describe for each layer and each cell cycle phase exactly how many cells were examined, total length of observation time and the number of transitions observed out of that phase (lines 372-407). That is the entirety of the relevant raw data (the “actual data” referred to by the reviewer). We have now also added to the supplemental data table three additional spreadsheets for Fig. 4 with the raw data of time imaged for each cell in each cell cycle phase from each imaging session, together with recorded transitions, for WT, excess CycE and excess Hop (tabs of Fig4-WT, Fig4-UASCycE and Fig4-UASHop).

The issue of logic and understanding is entirely separate from a search for raw data (the data are all in the text, which explains how it is used to calculate the time spent in each cell cycle phase). The reviewer is asking about two issues.

1. Calculation of cell cycle times from live imaging data.

Here I am just re-phrasing the written text, with a numerical example, in the hope it might be clearer. We are sampling roughly 2h periods of time for many germaria. This generally does not capture a cell entering a specific phase (say G2), residing there, and exiting (to M and then G1). However, we sample many equivalent cells (same genotype, same AP location). Each sampling period begins at a random time relative to when a cell may have entered the phase we see at t=0. Imagine, for example, that G2 lasts 5h on average, that every imaging period is 2h and we capture cells distributed evenly at 30min intervals into G2 at (30, 90, 150, 210, 270 mins). Over 2h, those captured at 210 and 270 mins might (on average) exit G2 during imaging. We would have 5 cells imaged for 2h each (total 10h) and found 2 that exit G2, so we deduce a G2 phase of 10/2= 5h. Although the example uses regular intervals and uniform passage through G2, the logic applies equally when each is variable.

Motivated by the reviewer comment, we have also added a diagram and short mathematical explanation for the general case of any sampling period, x, and cell-cycle phase period, y, within a cell cycle of length z as a supplementary method.

2. Why EdU indices do not suffice to report relative cell cycle times.

This is a straightforward issue that comparing two ratios (EdU indices) is insufficient to deduce the ratio of the two denominators (total cell cycle time) if the two numerators (time in S-phase) might differ.

Our EdU measurements are made over a period of 1h immediately after dissection. For the sake of simplicity (the logic is unchanged), let us imagine that the measurement is at an instant (1h is very short compared to the minimum cell cycle times we are measuring) and it is simply reporting whether each cell is in S-phase or not. The probability that any one cell is in S-phase equals the duration of S-phase divided by the total cell cycle time (E1=S1/T1 and E2=S2/T2). So, for example, if we score 50 wild-type cells in layer 1 we would likely find about 17 are EdU-positive and the probability of being in S-phase is 17/50 (34%). If an altered genotype shows an EdU index in layer 1 of 17%, then we can deduce that (mutant S-phase length)/(mutant total cell cycle time)= 0.5 x (WT S-phase length)/(WT total cell cycle time). We cannot deduce anything about the relative total cell cycle times unless we know something about relative S-phase durations (T1/T2= (E2/E1)x(S1/S2)). If we knew that S-phase duration was constant (we do not), we could deuce that the mutant cell cycle time is twice as long as for wild-type. Because G1 and G2 are classically considered to be regulated, some may inappropriately assume S-phase is constant (in different locations and genetic conditions for FSCs). Our studies show that assumption would definitely be incorrect. We do not spell it out, but exactly the same logic applies to measuring the proportion of cells in M-phase. The key virtue of live imaging is that we see transitions over time and do not therefore need to make invalid assumptions.

In the revised text, this is explained in lines 354-361

The next data issue is the new technique of H2B-RFP dilution. It is a new assay using new reagents, yet it is not described or validated sufficiently for the reader to have confidence in it. The all-off (18°) and all-on (29°) conditions need to be shown in a supplement. The authors note that “the pattern of labeling intensities among different cells were very similar to those of GFP from a UAS-GFP transgene present in the same animals” but that data is missing.

We have added images comparing UAS-GFP and UAS-H2B-RFP expression in the same germarium as Fig. S2.

There are a great many qualitative statements without quantitative data to back them up. The most problematic is this: “Under all tested conditions, we observed uneven H2B-RFP (and GFP) intensities among germarial cells”. Without providing evidence about the distribution of these uneven intensities immediately after labeling, we cannot evaluate the significance of any change observed later that is attributed to dilution – we need the variability of both the starting conditions and the later dilution conditions, and we need to see if any difference in these highly variable numbers are statistically significant. This analysis will require not just chi-squared tests, comparing observed to expected, but statistical tests that will consider data variability. Other vague statements: “H2B-RFP signal was largely cleared…” [please show and quantify], “FSCs retained a strong H2B-RFP signal, while intensity in some layer 1 FSCs was markedly lower but clearly detectable” [how many layer 1 FSCs?].

We presented observations in two ways to take account of variability in H2B-RFP intensity among FSCs in a given layer. First, we presented data on the average H2B-RFP intensity for each FSC layer over time. Second, to give an idea of the frequency with which the trend of average intensity changes over time was upheld by individual cells, we reported the number of cells per layer with H2B-RFP intensity below a given threshold (4-fold reduction).

The results are explained in qualitative terms together with clear explanation of the limitations.

The qualitative conclusions were supported by all samples examined and illustrated in Fig. 5A-F, which are representative images. It is clear that H2B-RFP signal remains strongest during chase in ECs, then layer 3, then layer2 and then layer 1 FSCs (with FCs even weaker). Differences between individual cells in a layer are also readily seen from these images.

Systematic reasons (including FSCs moving between layers) why the methodology cannot report division rates in a given location are also discussed.

We have altered the text to make it even clearer (with precise statements) that we are making limited claims from these data, describing and showing the underlying data.

We believe the reviewer is asking for proofs beyond the systematic limitations of this technique, as applied to FSCs.

Smaller points:
• In Fig. 1A, indicate R1 and R2A. Also R2a and R1 are capitalized in Fig. 3 but never in the text; standardize them.

Done
• Show the expression pattern of C564Gal4 in a supplementary figure. Its gradient is discussed in the text several times, and it is a key reagent.

We have added two panels showing C587-GAL4 expression as A and B in Fig. S1 (referenced on line 138).

• In Fig. 6A, the diagram is confusing, and the legend does not help sufficiently. Same for the numbers above the chart in panel D – please label the 4 different numbers on the figure because the legend is very confusing (same with the similar charts in Figs. 7,8,9)

We have altered the main text and the Figure legend to describe the numbers above the graphs in Fig. 6D at greater length.

I am concerned that the reviewer refers to Figs 7,8, 9 as MARCM experiment data. The revised manuscript eliminated most MARCM studies (those of the old Figs 7-9). If the reviewer was in fact reviewing the original version of the manuscript, that would likely contribute to the impression of the manuscript being very hard to read.

Reviewer #2: In this MS, the AUs report a thorough analysis of the cell cycle of live FSCs utilizing the FlyFUCCI tool and other genetic manipulations. They address the proliferation differences found in the 3 FSC layers and investigate the nature of the signals that may generate the diverse proliferative behaviors found in the FSC population. The AUs have made a significant effort to assemble a more tractable and easier to read MS (compared to the original submission). Still, it is considerably dense and detailed in its descriptions and analyses. In my opinion, the data presented are rigorous and comprehensive, and accompanied by pictures of great quality.
1- My main concern is the adscription of colorless (non-GFP, non-RFP) cells to the S-phase. In the text, the AUs state that “cells with GFP-only or GFP-plus-RFP expression did not include EdU, consistent with assignment of all such cells to G1 and G2 (or M), respectively (Fig. 1C-H). Some EdU-positive cells included RFP but the majority were colorless, showing that the delay in accumulating RFP in S-phase is extensive. All RFP-only cells had EdU label, indicating that GFP accumulates quickly at the onset of G2. Thus, G1 (GFP only), G2/M (GFP + RFP) are clearly defined, while S-phase cells can be recognized as lacking both GFP and RFP”. There is however at least one precedent in which other AUs conclude that non-RFP non-GFP FLyFUCCI cells are either in G1 or S phase (Villa-Fombuena et al, Dev. 2021). So my question for the AUs is whether there are colorless cells that are EdU negative? And in what frequency? This is an important point, particularly so considering the number of observations in which they quantify cell cycle phases considering these cells as S-phase.

In addition, the very short G1 phase compared to the long S-phase described for most of the cell types and experimental situations (see for instance Fig. 4C) is striking when compared to other stem cell types.

The question is whether we are correct in assigning colorless cells to S-phase.

We cannot give a direct answer to whether every cell without GFP or RFP had EdU because we could only use three fluorescent channels and therefore stained for EdU or DAPI, but not both. When we used DAPI on WT samples, we found that the sum of colorless and RFP-only cells (putative S-phase cells) was very similar to the proportion of EdU positive cells scored over a very large number of MARCM experiments (33% vs 33% layer 1; 24% vs 20% layer 2). This is stated directly after the quoted passage as support for EdU labeling or GFP-negative FUCCI being equally good measures of S-phase frequencies. We have elaborated that section (lines 159-168) to (i) include the actual numbers of colorless plus RFP-only cells in layers 1 and 2, and (ii) to explain that the correspondence of S-phase cells determined with and without EdU is consistent with there being no mystery cells- all cells have GFP, RFP or EdU signal, and when EdU is not used, the phase of all cells can be determined by a combination of DAPI, GFP and RFP.

Later in the text, we note that during live imaging GFP-only cells always lose GFP without immediately gaining RFP signal. We also note that the number of RFP-only cells in layer 2 substantially exceeds the number of RFP-only cells in layer 1, even though many more layer 1 FSCs are EdU-positive than layer 2 cells. These observations are entirely consistent with colorless cells being in S-phase and only transitioning to RFP-only cells after a substantial time in S-phase (with layer 2 cells spending a much longer time in S-phase and therefore, on average, having a much higher RFP: colorless ratio than layer 1 cells).

We believe the explanations in the revised text are sufficient to clarify the evidence and conclusion that colorless cells are in S-phase.

There are additional arguments, which we considered carefully but did not put in the text for brevity.

The most likely systematic reason for the absence of GFP or RFP in a cell cycle phase that simplistically ought to express GFP or RFP is that the marker has been degraded in the prior phase and has to accumulate de novo before being visible.

For GFP, this would happen directly after S-phase and a delay would mean that an early G2 cell might have only RFP. However, as stated, all RFP-only cells were labeled by EdU. Hence, all RFP-only cells are in S, not in G2. The result also shows that expression of GFP must be very robust in our genetic set-up to avoid delayed detection.

For RFP, a delay in accumulation after RFP loss during G1 following mitosis would lead to S-phase cells that are colorless. In general, RFP signals were much weaker than GFP signals (for example, in G2 cells), so delayed RFP detection at the onset of expression is highly plausible. It was also seen many times in live imaging that GFP-only cells became colorless; they did not ever directly become RFP-only. Moreover, it is certain that the vast majority of colorless cells are in S-phase, as explained by the numbers quoted above- very few layer 1 cells are RFP-only but adding colorless cells to RFP-only cells matches observed EdU frequencies.

The other potential “artifactual” reason for a cell being colorless is simply that expression levels of GFP and RFP are lower than ideal (exacerbated at the times they must first accumulate, as explained above, but potentially sporadic at any time if expression is not robustly above the threshold for detection). For this reason, we tested out several drivers, settled on C587-GAL4 and always used two copies of UAS-FUCCI in order to achieve robust expression. As explained above, this was clearly successful for GFP because there was no delay in GFP appearance after S-phase. Hence, it is very unlikely that any cell in G1, which has been expressing GFP for a very long time, would fail to exhibit a GFP signal simply because of inadequate levels of expression.

Finally, the GFP fusion is with a segment of E2F1. The FLY-FUCCI paper and references therein provide the theoretical reasoning why this fusion protein is very unlikely to be degraded during G1 and there is experimental evidence in favorable settings (wing discs and tissue culture cells) that it is degraded during S, not G1.

From all of the above, it is clear that the vast majority of colorless cells are in S-phase. It also seems implausible to me that any colorless cells are in G1 (because E2F1-GFP degradation in G1 is theoretically very unlikely and GFP signal strength is very robust and has been present in G1 cells for a long time).

We thank the reviewer for referring to the very interesting paper on using FUCCI for female GSCs. In that paper, as in our study, live imaging showed transitions from GFP-only through colorless to RFP-only. The authors then used spectrosome morphology in fixed samples to try to determine the phase of colorless (“black”) cells. “Round” morphology was seen in some GFP-only cells (early G1 from live imaging) with slightly more “Bar” plus very few “plug”. RFP-only cells were “Fusing”, as were some (GFP+RFP) positive G2 cells. The authors deduced that “Fusing” showed that a cell is in S (however, it could be that all the RFP-only cells had not yet acquired GFP and that “Fusing” is actually characteristic of G2). Of the colorless cells, similar numbers had “round”, “Bar” and “Fusing” spectrosome morphologies. The authors assigned the first two to G1 and the latter type to S. However, (a) there is no guarantee that the spectrosome cycle is synchronized to G1 or S (it could equally be dependent on time since mitosis or lacking any strict synchrony) and (b) there is no sound reason to be confident that a G1/S transition (if synchronized to spectrosome morphology to some degree) corresponds to the “Bar”/”Fusing” transition. The authors did not use EdU labeling or discuss how plausible it is that E2F1-GFP is degraded during G1. I personally remain unconvinced that some of the colorless cells were indeed in G1. Among other possibilities, if overall GFP and RFP expression was not robust, colorless cells could also be in G2, with a “round” G2 spectrosome morphology attributed to G1.

Altogether, the paper does not provide unequivocal evidence for a systematic limitation of FLY-FUCCI of surprisingly premature E2F1-GFP degradation, leading to colorless G1 cells, while evidence within our system shows that to be very unlikely and assignment of colorless cells to S is entirely consistent with logic and correspondence to EdU indices.

2- Another general comment. I believe it would help assess the significance of the findings if the AUs were to indicate the sample size in the different quantifications. Perhaps not for all of the genotypes or experimental conditions, but to cite a range (for instance, n>20 for all of the genotypes) and perhaps not in the text or in the fig. itself, but in the fig. legends?

This information has been added to the legends of Fig. 1, Fig. 3 and Fig. 6. It is also listed in detail in the supplementary data table for each Figure. The precise numbers for the summary figure in Fig. 4C are already in the main text (and are now also broken down for individual cell measurements in supplementary spreadsheets).

3- The text citation of Figs. 2, 3 and 4 is rather confusing. The citation order in the text is the following: figs. 2 + 3 (line 177), 3A-C (191), 4 (194), 2A + 3 (205), 2B + 3 (215), 3D (225), 2D + 3A-C (234), 2E + 3A-C (256)… I have struggled a bit to follow the text and the corresponding figs. This could be improved, perhaps by reorganizing Figs 2 and 3 and moving the EC data to a new Suppl fig.?

I appreciate that the reviewer is seeking to improve clarity. However, the Figures are arranged quite logically in my opinion. Fig. 2 shows a series of illustrative images for a given genotype and Fig. 3 shows the numerical data. It is therefore common to refer to both Fig 2 and Fig 3 for a specific result where images are shown. We choose to put all of the quantitative data from the same type of experiment together because it allows multiple comparisons (horizontally and vertically). Some of these comparisons are spelled out in the text with numbers but readers might like to make their own side-by-side comparisons. The comparisons can be genotype to genotype across a row or among different cell types and locations along a column. Although ECs may be considered less central than FSCs, we would lose the opportunity to compare readily between EC and FSC responses if we presented EC data separately. We struggled to find a format to allow fitting these data on to one page and think we (just) found an excellent solution (including a very concise Figure legend). There is indeed a wealth of data here, but we are providing a full, transparent and logically arranged summary. We also chose the images and genotypes carefully to feature the characteristic phenotype relatively easily.

4- Line 101: Please introduce the concept of FSC depletion. Is there a Ref. for the 4-fold higher depletion rate of layer 1 FSCs?

This is now explained more fully and referenced in the Introduction, along with a quick summary of the evidence for FSC locations (lines 84-102).

5- Line 401: See my comment above RE colorless and S-phase.

Response described earlier.

6- Lines 662-664: “Live imaging with FUCCI reporters allowed us to record the frequency of cell-cycle transitions and deduce absolute cell cycle times. These parameters have not previously been measured for FSCs and are generally not known in other adult stem cell paradigms.” You may want to consider citing here Villa-Fombuena et al. These AUs precisely utilize FlyFUCCI to look at female Drosophila germline stem cells in live ovaries.

We have added that citation in an added sentence.

Reviewer #3: In this revised paper the authors examine the cell cycle regulation of FSC layers, which give rise to both Escort Cells and Follicle Cells, which have distinct cell cycle parameters, numbers and positions. They use FUCCI imaging (both live and fixed analysis), EdU labeling (with 1 hour labeling intervals) Histone-RFP depletion assays (to assess proliferative heterogeneity in FSCs) and MARCM clonal analysis to examine how cell cycle and signaling pathway manipulations impact the cell cycling parameters of FSCs. In brief they uncover: heterogeneity in the FSC cycling population such that layer 1 (giving rise for FCs) cycles fastest with a significant fraction in G2, layer 2 more slowly and layer 3 most slowly. They establish signals (specifically Jak/Stat and PI3K, with lesser data on Hippo signaling) that impact these transitions and identify CyclinE and Stg as the most likely downstream cell cycle machinery modulated by these signaling pathways.

One limitation of this story is that much of the evidence for the epistasis is quite indirect and thus the pathways are left somewhat unclear. For example, Dacapo over expression partially limits the cell cycle effects of Hop OE, but it is not clear whether Hop OE increases CycE expression, impacts Stg expression, or how exactly it affects G1 and (mostly) G2 timing. Similarly, the clonal analysis via MARCM shows partial rescue of Stat loss and cell cycle arrests by overexpression of CycE and Stg, but clearly there are unaddressed feedback loops that make the epistasis, even with CycE and Stg overexpression, inconclusive (e.g. adding CycE and Stg cannot fully restore cycling in the absence of Stat, which the authors point out). In addition, the regulation upstream or downstream of PI3Kinase activity and the Hippo pathway is unknown/unaddressed beyond the most downstream cell cycle regulators CycE and Stg. Altogether this provides detailed data on how specific genetic manipulations alter the cell cycle in FSCs, but does not focus on a specific pathway, and leaves unclear what exactly PI3K, Jak-Stat and Hippo pathways are doing to modulate FSC cell cycling. That said, I recognize there are limits to any one study and there has already been significant revision to shorten the paper. I therefore will try to limit my comments for further revision to suggestions to streamline the story or clarify issues.

In our original manuscript, we included many more MARCM lineage experiments, some aimed at investigating epistasis and therefore testing a small number of mechanistic hypotheses. Causal molecular interactions are very challenging to discern and prove, especially when addressed physiologically and centered on cell cycle regulation, which has numerous potential inputs and internal feedbacks. Our initial genetic tests of potential molecular mechanisms were therefore put aside, with the advice of reviewers, in order to focus on the major insights afforded by developing fixed and live FUCCI imaging for FSCs. I believe our paper has very important insights about absolute cell cycle times that are integral to the organization of FSCs and on how (at an over-view level) JAK-STAT signaling, and to a lesser extent, the PI3K pathway, affect transitions between cell cycle phases (and overall cycling times for JAK-STAT). We do not attempt to make mechanistic molecular connections at all. The use of Cyclin E and String is for the purposes of targeting G1/S and G2/M transitions, respectively, in order to compare to the effects of signaling pathways and to test which transitions are limiting for ECs and FSCs in different locations. Even in the limited epistasis tests presented, we are not inferring that *cycE* or *stg* are direct transcriptional targets of JAK-STAT signaling. We are instead using those genes to provide evidence about deficits in specific transitions when STAT activity is lost. I would therefore agree with the reviewer’s opinion that there are mechanistic questions that will probably be difficult to figure out even over a number of subsequent publications and that the positive content of this paper is of a different nature (acknowledged but not spelled out by the reviewer).

One strength is that the authors carefully address the limitations of each approach to measuring the cell cycle parameters, which I appreciate. For example, fixed imaging approaches fail to capture dynamics and fail to accurately measure lengths of cell cycle phases. However a fundamental flaw in the live imaging of FUCCI cells is the loss of color during S-phase leading to an inherent inability to track cells throughout the cell cycle. Thus both approaches must be used together to confirm the cell cycle parameters.

There is no flaw or deficit in figuring out total cell cycle times for live imaging (as the reviewer seems to imply). By following cells in G1 or G2 and how often they exit that phase, we directly deduce the average duration of G1 and G2. We could use either one of those numbers to calculate the overall cell cycle time because we know the proportion of cells (of a given type) in G1 and G2 (from both fixed and live imaging). We choose to explain this in the text by adding the times spent in G1 and G2, as well as the fraction of a cell cycle occupied by G1+G2 (because the errors will be smaller for a larger denominator) and to first explain how S-phase length is deduced (before going to total cell cycle time). There is no deficit in the data for the fraction of cells in a given cell cycle phase from fixed images, and hence no detriment from combining the live imaging and fixed imaging data.

A limitation of the H2B-RFP dilution imaging was an inability to equalize starting amounts of H2B-RFP expression prior to tracing. The authors properly address each of these limitations (which is appreciated since these are often ignored),

Thank you

(the rest of the sentence appears to be circling back to FUCCI studies)

and use a combination of estimates from each cell cycle transition and its frequency to arrive at estimates of cell cycle phase timing for layer1 and layer 2 FSCs that roughly recapitulate estimates from fixed imaging. (They are unable to measure dynamics for layer 3 FSCs.) They uncover interesting differences in S-phase length between layers of FSCs, (which might suggest differences in E2F activity and therefore differences in levels of DNA synthesis/repair targets). This could possibly be addressed by looking at reporters of E2F transcriptional activity. In addition, I wonder if the regulation of PI3K activity could be addressed by looking at the tGPH reporter.

We were indeed unable to measure layer 3 FSC cycling time but our evidence confirms very slow cycling. Hence, the exact magnitude is unlikely to be of great consequence.

How S-phase may be regulated is, again, an interesting but extensive subject for further investigation. Likewise, we have already discarded data from further genetic investigation of PI3K actions and, as explained in initial review responses, did not find strong enough signals from existing tGPH reporters to conclude anything about the pattern of PI3K activity.

The H2B-RFP imaging description is complicated, due to the caveats that the authors point out. In sum, it supports the general finding of cell cycle speed from the live and fixed FuCCI experiments and I suggest it could largely be put into the supplement (and only briefly summarized as additional evidence supporting the FUCCI study) to address the length and complexity of the current (revised) manuscript. The authors describe the importance of this section being due to the evidence of FSC movement between layers - but I would suggest this portion showing FSC movement can be retained in the main manuscript, but without the additional details of the H2B-RFP dilution dynamics to simplify.

We appreciate this suggestion. However, another reviewer requested more information about the quantitative aspects of the H2B-RFP studies. My opinion is that images generally convey important information succinctly and without embellishment. Also, most readers are not inclined to look at supplementary materials. I therefore believe it is important to retain the limited (six) panels of Fig. 5A-F, which concisely illustrate the major findings regarding H2B-RFP dilution. The descriptive text has been shortened and clarified to retain critical numbers in a form that is more readily understood, and to explain the limitations on quantitation. We also include a clearer transition from the virtues of H2B-RFP experiments that are complementary to those of FUCCI tests towards the use of those samples to observe FSC movements during live imaging.

Clarification points:

Excess Cyclin E prolonging of S-phase can cause DNA damage and replication stress (see <https://www.frontiersin.org/articles/10.3389/fcell.2021.774845/full> and references therein). Is DNA damage incurred in FSCs in the cell cycle manipulations and thus reducing the subsequent proliferative capacity of FSCs when S-phase is prolonged? As mentioned above, if the signaling environment impacts the levels of E2F activity in each FSC layer, this could alter how the cells respond to CycE and Stg manipulations.

We thank the reviewer for the reference. We find that excess CycE alone speeds FSC cycling modestly and leads to net FSC accumulation over time. This was also true when combined with loss of STAT function (with or without additional alterations to Stg or kibra). S-phase duration was only modestly extended by excess CycE. Thus, under these conditions, there is presumably no drastic DNA damage, though, of course, some replication stress may limit the CycE responses. Moreover, excess CycE is less effective in more anterior locations where all cell cycle phases are lower. We therefore see little reason to investigate replication stress or DNA damage as a likely factor limiting productive responses to CycE.

The condition of excess CycE together with Stg in MARCM studies, however, clearly gave very high EdU indices but slightly reduced FSC accumulation. Here, there is clearly a detrimental response of some sort and we suggest replication stress. We did not, however, look directly for indicators of this.

Page 86 line 199 - the minimal effects of CycE OE in anterior domains could be due to limiting Cdk2 levels (as opposed to different CycE/Cdk2 thresholds as the authors propose). The authors could examine this by co-overexpressing CycE and Cdk2 or could explain both possibilities.

We have amended the text to include this specific possibility (line 226).

The experiments blocking the Hippo signaling pathway in Stat mutant clones, with and without CycE/Stg expression seem to lack some context to explain how it fits into the revised story. If I understand correctly, the sum total of this experiment is that CycE & Stg are downstream of both pathways (already known) but that Hippo signaling and Stat loss still limit the level of FSC cycling that can be induced by CycE/Stg (e.g. effects of CycE/Stg expression are stronger when Stat is present but are partially enhanced when Kibra is also lost). Overall this suggests a non-linear pathway with additional targets affected by both Hippo and Stat signaling outside of CycE and Stg that feed into the response to the ectopic CycE and Stg. Should this and potential additional cell cycle regulatory targets of these pathways be discussed?

We appreciate the reviewer’s interest in the subject matter. We would eventually like to figure out some mechanistic details, at least to a first approximation. In the original submission we include much more MARCM data relevant to the Hippo pathway, partly to re-examine the connection between Hh signaling and yki, and to test for an analogous connection between JAK-STAT signaling and yki transcriptional induction (the results indicated a connection but also actions of JAK-STAT independent of yki induction). All of that is not really relevant to the single mention of using kibra mutations in MARCM tests. The purpose here is simply to raise the EdU index of stat mutant cells to provide another setting to test for enhancement by excess Stg. We offer the background that previously published studies suggest Yki acts principally by stimulating cycE expression but even that is not strictly necessary to simply test a response to UAS-Stg. In short, we are certainly not investigating the Hippo pathway in this paper, nor (as explained earlier) is there an objective to define molecular connections more generally.

This is outside the scope of this paper, but I’m very intrigued by the stimulation of S-Phase entry seen by excess Stg in ECs. This suggests some cell type-specific cdc25a/b function for Stg which is interesting. I wonder if these cells are entering an endocycle rather than a mitotic cycle?

**Have all data underlying the figures and results presented in the manuscript been provided?**
Large-scale datasets should be made available via a public repository as described in the *PLOS Genetics* [data availability policy](http://journals.plos.org/plosgenetics/s/data-availability), and numerical data that underlies graphs or summary statistics should be provided in spreadsheet form as supporting information.

Reviewer #1: **No:** see review and confidential comments to the editor

Reviewer #2: Yes

Reviewer #3: Yes

PLOS authors have the option to publish the peer review history of their article ([what does this mean?](https://journals.plos.org/plosgenetics/s/editorial-and-peer-review-process#loc-peer-review-history)). If published, this will include your full peer review and any attached files.


If you choose “no”, your identity will remain anonymous but your review may still be made public.


**Do you want your identity to be public for this peer review?** For information about this choice, including consent withdrawal, please see our [Privacy Policy](https://www.plos.org/privacy-policy).

Reviewer #1: No

Reviewer #2: No

Reviewer #3: No

Below are Editor comments (black) and our responses (blue).

As you will see in the specific comments of the reviewers, there is a mixed view on the suitability of this manuscript for publication in PLOS Genetics. In addition to the detailed feedback provided by the reviewers directly to the authors, they raised several points in their comments to the editors that are important to address and which I summarize below:

1) All three reviewers found the manuscript an exceptionally difficult read, and they expressed concerns that this would lessen the impact of the work after publication. One reviewer suggested that the MARCM section of the manuscript could be deleted; another suggested limiting the MARCM data to the JAK/STAT pathway (because of the lack of information on the spatial levels of PI3K and Hippo signaling). This reviewer also suggested moving much of the H2B-RFP dilution data to the supplement. Besides concerns about the clarity/accessibility of the manuscript, two other concerns in the reviewers’ comments to the editor were as follows:

The MARCM section is already limited to JAK-STAT (that reviewer appears to have been looking at the original manuscript version, which may underlie or exacerbate the general comments about a difficult read). We altered and justified our presentation of H2B-RFP studies in specific responses to reviewers. That section makes an interesting addition, with key bottom-lines clearly illustrated visually by a single main figure and supplementary movies. In addition to a large number of clarifying text re-writes that were spelled out in specific responses to reviewers, many additional changes were made throughout (especially in sections regarding fixed FUCCI data with different genotypes and MARCM test) to eliminate, simplify and clarify.

I believe the structure of the paper is logical and straightforward with clear take-home messages throughout, as laid out below. Each method and the logic of conclusions are spelled out carefully with due introspection and careful description of data shown in Figures.

1. FUCCI method with fixed samples: G1, G2 as designed; many S-phase cells have no RFP.

2. Wild-type FUCCI analysis: strong anterior-posterior gradient of G1/S regulation

3. Genetic changes with fixed FUCCI analysis: JAK-STAT promotes G1/S and G2/M transitions in FSCs & unlike CycE also promotes G1/S in ECs. PI3K promotes G2/M, Wnt has no effect.

4. Live FUCCI method & results for wild-type: absolute cell cycle times measured for first time. The numbers confirm productivity of FSCs matches differentiated cell output. Posterior FSCs cycle 3.4-fold faster than anterior FSCs, producing enough cells to match FC output, while anterior FSC division matches EC output. Hence, exchange between layers is at equilibrium.

5. Live FUCCI with excess CycE or JAK-STAT: JAK-STAT speeds cycling much more than CycE despite similar EdU indices (because S-phase length differs). Flattening the JAK-STAT gradient makes posterior and anterior FSC cycling more similar but still >2-fold different, consistent with another, unknown graded division signal.

6. H2B-RFP dilution method & results: qualitative confirmation of FSC proliferation gradient, used also for live imaging to show movement of FSCs in both directions directly for the first time.

7. MARCM lineage studies: confirm that excess JAK-STAT promotes proliferation more than excess CycE through greater accumulation of marked FSCs despite a higher differentiation rate. Confirm actions of JAK-STAT on G1/S and G2/M through complementation of STAT loss only by combining excess CycE and Stg.

The sum total of these results is a major advance regarding technical possibilities (measuring absolute cell cycle times) and full understanding of FSC behavior (balance of division and differentiation accommodated within starkly heterogeneous properties of individual stem cells through dynamic exchange at equilibrium). Both the consequent understanding of cycling (parameters and regulation) and overall organization (behavior by location) of FSCs compares favorably with any other adult stem cell paradigm. Hence, this paper should be of wide interest, especially to those wishing to understand adult stem cell biology.

Although reviewer comments were dealt with exhaustively above, the major issues were clearly:

Reviewer 1: Skepticism that FSC locations can be reliably identified fully answered (together with reference to background of justifying locations of FSCs through functional assays). Unfounded claims of missing primary data coupled to mis-understanding of methods for determining cell cycling rates: both carefully explained and further documented.

Reviewer 2: Doubts over the identity of colorless cells as being in S-phase: answered extensively in revised text and additional discussion.

Reviewer 3: Concern over colorless cells limiting live FUCCI deductions: explained that this is a false concern. Interested substantially in mechanistic cell cycle questions that are beyond the scope of this paper.

Not only have all the major and minor issues been answered, but also the detail and volume of comments has led to revisions that include extremely careful language, documentation and full explanations.

2) The reviewer who raised concerns about the spreadsheets for Figure 6 was motivated to look at the raw data to learn more about possible variability in the percent positive Edu cells between each germarium. Providing more information in this regard would be helpful.

This reviewer did not spell out this particular concern in comments that we saw (a large number of other very exacting comments were fully answered in great detail). As phrased in the Editor’s summary, I cannot respond fully because I do not understand the comment. If it refers to EdU in MARCM tests, I cannot see what insights could be discovered by looking at variation. Our raw data are tabulated for each germarium as the number of GFP-marked FSCs in each layer and the number of those FSCs which are EdU-positive. Typically, there are about 3 GFP-positive FSCs in a wild-type germarium (about half in layer 1) on average and the EdU frequency in layer 1 is about 30% on average. So, the individual EdU labeling frequencies for layer 1 FSCs (when present) will mostly be in the range of 0-3 out of 1-3 cells (seeing roughly one out of three is quite common). These are not the sort of data that can report meaningful variation.

3) One reviewer was surprised there was no mention in the manuscript concerning the controversy between your published work and that of Todd Nystul. Would be a service to readers to discuss this briefly in the introduction.

I think it would not be a service to readers to discuss this issue at length. The politics surrounding this issue (with many improper practices, in our opinion) has been an unnecessary and inappropriate blight on perception of our research for several years now. Our studies in 2017 and all of our publications since then, including this one, provide compelling, clear and consistent evidence of the FSC arrangement and behaviors we describe in the introduction. Dr. Nystul has never produced evidence or arguments that contradict our conclusions in a scientifically defensible way. This includes a refusal to entertain the possibility that FSCs could be maintained by population asymmetry, in deference to the original FSC model that assumed single-cell asymmetry, and it includes totally unjustified attacks on our methodology and conclusions (that escaped serious critical review) to which we were not allowed to respond in print (despite reviewer support). All of the salient science has been addressed previously in our research papers, a recent review and a bioRxiv commentary on false objections raised by Dr. Nystul. Rather than making a false controversy and its rebuttal central to this paper, we have (i) responded to reviewer 1 questions about FSC identity by explaining more about determination of FSC identity at the outset and (ii) given your general comment, we have added to the Discussion how our new measurements further confirm the organization of FSCs and are not compatible with the original FSC model, along with references to the key relevant papers, including a review that discusses the whole topic from a general stem cell perspective.

To enhance the reproducibility of your results, we recommend that you deposit your laboratory protocols in [protocols.io](http://protocols.io), where a protocol can be assigned its own identifier (DOI) such that it can be cited independently in the future. Additionally, PLOS ONE offers an option to publish peer-reviewed clinical study protocols. Read more information on sharing protocols at <https://plos.org/protocols?utm_medium=editorial-email&utm_source=authorletters&utm_campaign=protocols>

Please be aware that our [data availability policy](http://journals.plos.org/plosgenetics/s/data-availability) requires that all numerical data underlying graphs or summary statistics are included with the submission, and you will need to provide this upon resubmission if not already present. In addition, we do not permit the inclusion of phrases such as "data not shown" or "unpublished results" in manuscripts. All points should be backed up by data provided with the submission.

While revising your submission, please upload your figure files to the [Preflight Analysis and Conversion Engine](http://pace.apexcovantage.com/) (PACE) digital diagnostic tool.  PACE helps ensure that figures meet PLOS requirements. To use PACE, you must first register as a user. Then, login and navigate to the UPLOAD tab, where you will find detailed instructions on how to use the tool. If you encounter any issues or have any questions when using PACE, please email us at [figures@plos.org](mailto:figures@plos.org).

PLOS has incorporated [Similarity Check](http://www.crossref.org/crosscheck.html), powered by iThenticate, into its journal-wide submission system in order to screen submitted content for originality before publication. Each PLOS journal undertakes screening on a proportion of submitted articles. You will be contacted if needed following the screening process.

To resubmit, use the link below and 'Revise Submission' in the 'Submissions Needing Revision' folder.


We are sorry that we cannot be more positive about your manuscript at this stage. Please do not hesitate to contact us if you have any concerns or questions.

Yours sincerely,

Ken M. Cadigan
Academic Editor
PLOS Genetics

Gregory P. Copenhaver
Editor-in-Chief
PLOS Genetics
