# Supplementary material for: Correction: Spatial regulation of Drosophila ovarian Follicle Stem Cell division rates and cell cycle transitions
Source: PLoS Genet. 2023 Nov 13;19(11):e1011040. doi: 10.1371/journal.pgen.1011040 (PMC10642776; doi:10.1371/journal.pgen.1011040)
Supplement: S1 Methods — (PDF) [file pgen.1011040.s001.pdf]

Live FUCCI Calculation Method

Here we present the method that was used to estimate the mean duration of cell cycle phases from multiple imaging periods of different cells for a period shorter than the cell cycle. We present (A) an intuitive perspective that may help with visualization, (B) a mathematical derivation based on consideration of probabilities, and (C) a set of illustrative examples to test the method. In each case, we consider first the simplest situation, where all cells imaged have identical lengths for each cell cycle phase. We then explore the effects of variation among cells of the duration of the specific phase being measured, of the total cell cycle time, or both. "Sampling Considerations for Live FUCCI Calculation Method" then explores the general validity of the method from a statistical sampling perspective and provides estimates of error margins for the data used in this work.

(A) Intuitive Perspective

Imagine there are a hundred identical cells, each of which is imaged for exactly one whole cell cycle. The duration of a specific phase (say, G1) can be calculated by dividing the total time cells were observed in G1 by the number of observed exits from G1 (in this case, 100). Imagine now that the same set of cells were imaged for only a couple of hours each, much less than a full cell cycle. In each case, you would be collecting data from just a segment of the cell cycle (let's say about 10%). There should be no systematic bias regarding the location of the segment (relative to any specific point in the cell cycle) and if enough samples are collected (100 in this example), they should collectively provide roughly even coverage of the common cell cycle (in this example, roughly ten-fold coverage overall). Collectively, therefore, these segments of the cell cycle could be put together in a way that reconstitutes roughly 10 circles, each with one exit from each phase, and with similar lengths of individual phases (this is not necessary but is just for the convenience of imagining circles similar to the true cell cycle), like a set of jigsaw puzzles. The sum of all of the times observed in a specific phase (in these 10 assembled circles or directly from the raw data without assembling any circles) divided by the number of exits from that phase should again, as in the first example, give a good estimate of the duration of that cell cycle phase. The quality of the estimate will depend on the density of sampling. In this thought experiment the total imaging time was about 10% of the full cell cycle from 100 cells. For wild-type layer 1 FSCs, the sampling density was similar to this illustrative example (89 cells tracked in segments about 15% of the mean cell cycle time [148 min periods on average, cell cycle mean estimated at 948 min]). The same method can be applied independently to estimating the average time for G2 or any other phase that is faithfully visualized.

If the population of cells imaged did not all behave identically, then the above method still has the potential to yield an accurate estimate of the mean length of each cell cycle phase, provided each cell behavior is sampled equally. This is considered more carefully in the mathematical treatment and illustrative examples.

(B) Mathematical derivation based on probabilities

Imagine that the length of a specific cell cycle phase, say G1, is “y”, that the whole cell cycle length is “z” and that the sampling (imaging) period of a cell is “x”. First consider a uniform cycling population of cells (constant y and z). The sampling periods could be drawn as segments on a large number of circles, each representing one cell. Instead, in the diagram below, the sampling periods are projected on to a single cell cycle and, for ease of drawing, the circular cell cycle is linearized (so, there would be identical representations either side, and a sampling period could extend into the previous or following cycle, just as for a circle). The sampling periods are drawn as evenly spaced and with specific alignments to the illustrated G1 period in order to illustrate specific length measurements clearly.

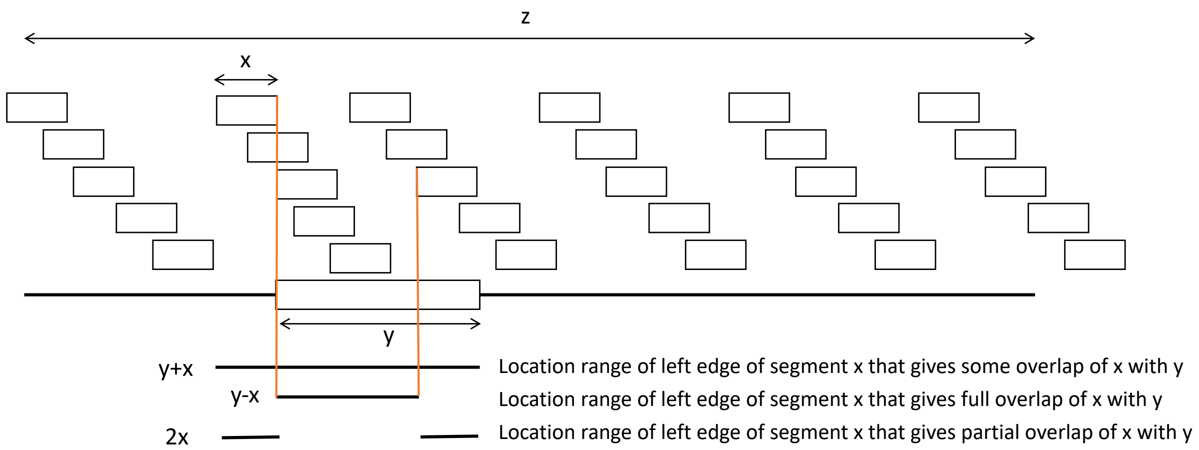

The probability of visualizing exit from G1 in a single sample (one imaged cell) is the probability that a length x overlaps any single point within the territory of length z, = x/z
If N cells were monitored, then the expected total number of G1 exits = Nx/z

The expected time a cell will be observed in G1 depends on the probability that the imaging sample overlaps with G1 and the time spent in G1. Here, there are two situations to consider- G1 residence during the entire imaging period or only for part of the imaging period.

A cell will be observed in G1 throughout the period x if the start of x is anywhere from the start of G1 to a point at distance x from the end of G1 (a span of y-x). So, the probability of partial residence in G1 during imaging = (y-x)/z.
The time in G1 in these cases would be the entire imaging period, x.

A cell will be in G1 for part of an imaging period if the imaging period starts at any time up to x before G1 (a span of x) or if it starts at any time within the last segment of length x of G1 (a span of x). So, the probability of partial residence in G1 during imaging = 2x/z
On average, the amount of time resident in G1 during such partial overlap incidents will be x/2 (by symmetry.

The expected time for a cell to be observed in G1
= (probability of full residence).(average time of full residence) + (probability of partial residence). (average time of partial residence)
$= [(y-x)/z].x + [2x/z].(x/2) = x(y-x+x)/z = yx/z$
If N cells were monitored, then the expected total time of cells observed in G1 = Nyx/z

If sufficient cells were imaged to achieve uniform, even sampling of cell cycles, then experimental observations should match expectations based on probability.
Then, observed total time in G1, "T"= Nyx/z and
The total number of exits observed, "E"= Nx/z.
Hence, $y = T/ (Nx/z) = T/E$.

The illustration uses an imaging period shorter than the cell cycle phase being investigated.
If the reverse is true, the probability of viewing an exit in one imaging period remains at x/z.
The probability of the entire G1 period, y, falling within an imaging period is now $(x-y)/z$.
The probability that only part of G1 is in the imaging period is $2y/z$ and the average time spent in G1 for this scenario would be $y/2$.
So, the expected time in G1 for a single imaging period= $y(x-y)/z + (y/2).(2y/z)$
$= y(x-y+y)/z = yx/z$
This is exactly the same as for $x<y$.
So, in all cases, given uniform sampling, the mean length of G1 can be estimated as $y = T/E$

"Sampling Considerations for Live FUCCI Calculation Method" considers errors due to random sampling, as opposed to "uniform sampling" considered above.

Heterogeneous cell populations

The reasoning above applies to a uniform population of cells with identical cell cycle properties.

In the more general situation, where each cell has a different length of a specific cell cycle phase (y1, y2, y3 etc.) and of the whole cell cycle (z1, z2, z3 etc.), the analogous equations for probabilities in one imaging period will be
Expected time in G1 = $x.(y1/z1+y2/z2+y3/z3 \text{ etc.})/N$
Probability of seeing a G1 exit = $x. (1/z1+1/z2+1/z3 \text{ etc})/N$
We are interested in estimating the average of (y1, y2, y3 etc.)

There are two special cases.
(i) If cells all had the same total cell cycle time, z
Expected time in G1 = $(x/z)(y1+y2+y3 \text{ etc.})/N$
Probability of seeing a G1 exit = x/z
So, with even sampling of N cells, $T= (x/z)(y1+y2+y3 \text{ etc.})$ and $E= Nx/z$
Hence, estimation of the mean value for $y = (y1+y2+y3 \text{ etc.})/N = T/(Nx/z) = T/E$

(ii) If cells had variable z but constant y,
Expected time in G1 = $(xy)(1/z1+1/z2+1/z3 \text{ etc})/N$
Probability of seeing a G1 exit = $x(1/z1+1/z2+1/z3 \text{ etc})/N$
So, with even sampling of N cells, $T= (xy)(1/z1+1/z2+1/z3 \text{ etc})$ and $E = x(1/z1+1/z2+1/z3 \text{ etc})$
Hence, $y = T/x(1/z1+1/z2+1/z3 \text{ etc}) = T/E$

Thus, a correct mean length of G1 (or another phase) should result from this sampling method and calculation if either (a) the length of the cell cycle phase or (b) the total cell cycle time is constant among the monitored cells.

If both y and z are variable among the cells visualized, then
E/T = (y1/z1+y2/z2+y3/z3 etc.)/(1/z1+1/z2+1/z3 etc)
The result will not generally be the mean y value but it will still provide an approximation. The deviation will depend on the magnitude of variation among y and z parameters within the population. This is explored, together with results for constant y, z or both, in the worked examples below. For the applications in this study of Follicle Stem Cells, each selected population of cells is narrowly defined as being of the same type (FSCs) and in exactly the same AP location within a structure that has no known radial asymmetry (orthogonal to the AP axis). Hence, it is expected that variations in cell cycle characteristics are expected to be stochastic rather than systematic and are plausibly of limited magnitude.

(C) Illustrated examples of live FUCCI cell cycle phase length calculation and relevant parameters

Imagine for simplicity that each viewing period, illustrated by each rectangle on the first three lines is 60 mins.
It is important to the method (and reflecting reality) that each viewing period begins at a random point in any given cell's cycle. For simplicity, uniformly even sampling is illustrated by spacing the sampling periods at regular intervals, each displaced by 30 mins. Intersections with the start or end of cell cycle phases would normally occur with a variety of overlaps but those illustrated have spacings of 15 or 45 mins for convenience of translating diagrams to discrete numbers.

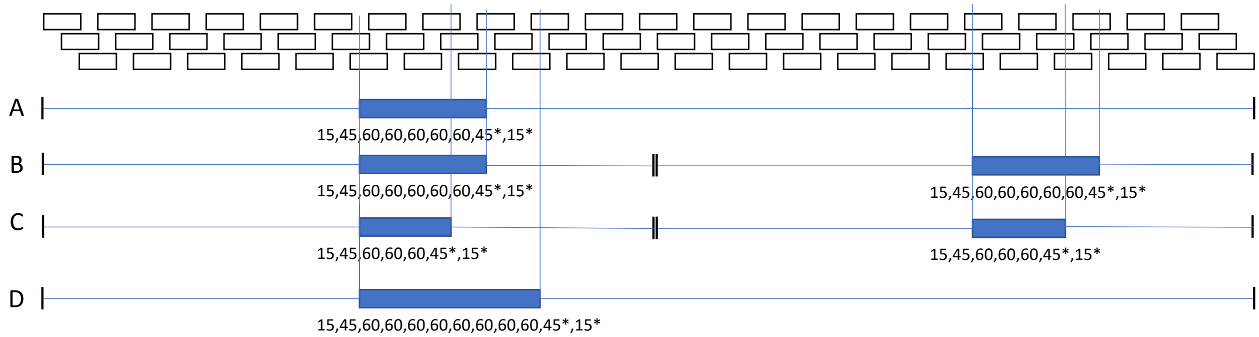

Lines A-D illustrate a variety of cell cycles with different parameters for cells named "A-D", with a specific phase, say G1, highlighted in blue. Calculations are first illustrated for homogeneous populations of A, B and C, followed by consideration of various mixtures of cells.

(a) If there were a population of cells, all behaving like cell "A", which were observed over the 66 illustrated 60 min viewing periods (one rectangle represents one viewing period of one cell), most viewing periods would not show a cell in G1 at all. The occasions when G1 is observed are when the viewing period rectangles overlap G1. For each of these nine rectangles, the length of overlap (in mins) is written (in order) beneath the blue rectangle, together with an asterisk if the viewing period includes exit from G1 (when the blue line vertical intersects the viewing period box). The total of time observed in G1 is 420 min with 2 exits. Hence, the estimated duration of G1 is 210 min. The blue rectangle is exactly 210 mins in length.

(b) Consider a situation where all observed cells are like cell "B", illustrated below A. Here, the length of G1 is also 210 mins but the length of the whole cell cycle is half that for "A" cells (half is chosen for convenience, but results would be no different for any other fraction chosen). Two cell cycles are drawn consecutively under the space represented by sampling periods, representing consecutive cycles. That is an appropriate way of showing the samples that would be observed for the same set of (66) 60 min samples illustrated for A. In situation B, G1 would be seen twice as often as in situation A (increasing the measures of both total time observed in G1 and number of exits from G1). The observed time in G1 for a set of B cells is written underneath each blue rectangle, together with asterisks for observed exits. Now, the total time observed in G1 over all B cells is 840 min with 4 exits. So, again, G1 length is correctly estimated as 210 min.

(c) Consider a uniform set of cells, C, with the same cell cycle length as B cells but a G1 phase that is shorter (drawn as 60 min shorter). For C cells, total time in G1 is 600 min with 4 exits, so the length of G1 is correctly calculated as 150 min.

In the examples above, there were either (a) two or (b, c) four exits observed. These low values are just to simplify diagrammatic presentation. In the experiments focused on wild-type layer 1 FSCs, twenty exits from G1 were observed (and 16 exits from G2).

(d) Consider a sample that includes both A cells and B cells. For simplicity, imagine that there are equal numbers of A and B cells, and that we now have twice the number of viewing periods (so that the same diagrams can be used directly). Total time observed in G1 will now be 420 min (from A cells) plus 840 min from B cells (sum of 1260 min). The total number of G1 exits will be 2 (from A cells) plus 4 from B cells (sum of 6). Hence, mean G1 length will be estimated as 1260/6= 210 min (again, correct). This illustrates that the method still works for a group of cells with different cell cycle lengths, provided G1 length is constant. That is in agreement with the mathematical derivation.

(e) Now consider an equal mix of B cells and C cells with 132 single-cell viewing periods, as above. Total time seen in G1 is 840 min (B cells) plus 600 min (C cells); total exits seen are 4 (B cells) plus 4 (C cells). Hence, the ratio (intended to give an estimate of mean G1 length) is 1440/8= 180 min. That is indeed the mean G1 length for an equal mixture of B cells (210 min) and C cells (150 min). This illustrates that the method still works for a group of cells with

different G1 lengths, provided total cell cycle length is constant. That is in agreement with the mathematical derivation.

(f) Now consider an equal mix of A cells and C cells with 132 viewing periods. Total time seen in G1 is 420min (A cells) plus 600 min (C cells); total exits seen are 2 (A cells) plus 4 (C cells). Hence, the method for estimating mean G1 length gives a result of 1020/6= 170 min. That is not the mean G1 length (180 min) for an equal mixture of A cells (210 min) and C cells (150 min), but it is not very different. This illustrates that the method does not work exactly for a group of cells with different G1 lengths and different total cell cycle lengths. The underlying reason is clear from the diagram. Cells, like A, with longer total cell cycle times will be sampled less frequently in G1 (if G1 lengths are reasonably similar), so the average G1 length calculated will be biased towards the faster-cycling cells. The sampling bias is not important for a mix of A and B cells (as in scenario (d)) because both contribute data for the same length of G1.
The error for an equal mix of A and C cells was 10 min/180 min = 6%. This error would be smaller if there were instead a distribution of cells with a range of properties bounded by the extremes of A and C.

The degree of variable sampling also depends on the relative lengths of G1 of different cells.
D cells illustrate a special case. Here, both G1 and total cell cycle time are double the values for C cells. From 132 viewing periods of an equal mix of C cells and D cells, total G1 time seen is 600 min (C cells) plus 600 min (D cells) and total exits from G1 seen are 4 (C cells) plus 2 (D cells). So mean G1 length is estimated as 1200/6= 200 min. That is not the exact correct value (225 min) for an equal mix of C cells (G1 of 150 min) and D cells (G1 of 300 min). Even though, the total time viewed in G1 was the same for C and D cells, the number of G1 exits was under-sampled for D cells. The estimated value of 200 min is (again) lower than the true mean (225 min) for G1.
The error for an equal mix of D and C cells was 25 min/225 min = 11%. This error would be smaller if there were instead a distribution of cells with a range of properties bounded by the extremes of D and C.

Since the scenarios of cells with different G1 and cell cycle times spanning a two-fold range of cell cycle times in the two examples of different G1 ratios (A plus C, or D plus C) would be less than 6% and 11%, it seems the error introduced by stochastic heterogeneity within a tightly defined cell population would be of a similar or smaller magnitude. Recall also that there is no systematic error of this kind if cells share either the same (or, in practice, very similar) G1 length (A plus B) or total cell cycle length (A plus D, or B plus C).

Might a relatively small population of fast-cycling cells obscure the properties of the major cell population when using total time spent in a phase divided by phase exits over all cells to estimate mean cell phase length?
Consider a population of 20% cells like C, together with 80% like A. The mean G1 length estimate would be (600+ 4x420)/(4 + 4x2)= 2280/12 = 190 min. The correct mean value would be (150 + 4x210)/5 = 198 min.

A similar scenario for 20% C plus 80% D cells yields an estimated mean of 250 min versus a true mean value of 270 min.

The two errors introduced by uneven sampling in these scenarios were 4% (8/198) and 7% (20/270).

These examples suggest that a small population of fast-cycling cells does not obscure the characteristics of the major cell population or greatly distort assessment of the true mean cell cycle phase time.

Might a small population of non-cycling cells obscure the properties of the major cell population?

The non-cycling cells may be arrested in G2. In this case, there would be zero impact on the estimation of mean G1 length of the cycling cells.

If the non-cycling cells were arrested in G1 they would add to the estimated mean G1 phase length (which may be appropriate if the non-cycling cells are considered part of the population). The magnitude of the addition would depend on the sampling period relative to the mean length of G1 (lower for shorter sampling periods). The sampling periods we use are relatively short (about 15% of the cell cycle for layer 1 cells and a much smaller proportion for layer 2). Hence, the numerical impact of non-cycling cells on mean phase length estimates will not be great.

Moreover, any impact can be sidestepped or lessened because we estimate mean G1 length and G2 length independently (from separate measurements). Cells arrested in G1 have no impact on mean G2 length estimation, and G2-arrested cells have no impact on mean G1 length estimation. So, one of the cell cycle phase length estimates is likely to be entirely unaffected by the presence of some non-cycling cells. Since we already know the proportions of cells in each phase from fixed FUCCI images together with EdU, the estimated mean length of G1 or of G2 suffices to estimate mean total cell cycle time. In this study, we used the sum of mean G1 and G2 length estimates to obtain the best estimate of mean total cell cycle time. We also compared the ratio of estimated mean G1 vs G2 derived from live FUCCI observations (81 min/527 min = 15.4%) to the ratio of cells found in these phases by fixed imaging (38/287= 13.2%) to see if one or other parameter might have been overestimated for a systematic reason, such as the presence of some cells arrested in G1 or in G2. The agreement between the two sets of G1/G2 ratios suggests there is no such major problem.

Summary

The method of calculation (total observed time in a specific phase divided by total number of observed exits from that phase from the same set of observations) used to estimate the mean length of a specific cell cycle phase from multiple segments of imaging of partial progress through the cell cycle can provide an answer with no systematic bias if the population of cells shares either a common phase length or total cell cycle length. A sampling bias that favors faster-cycling cells systematically reduces the estimated mean time when the population includes cells of heterogeneous lengths of both the individual phase measured and total cell cycle time. However, exploration of examples likely to resemble or overestimate the

heterogeneity encountered in a uniform cell population with stochastic variation suggest that the deviation is small (less than 10%) and that the estimation of the mean phase length is not very sensitive to the presence of some non-cycling cells.

The mathematical derivation and illustrative examples all assumed even sampling (starting an imaging period at any point in a cell cycle with equal probability). This assumption is strictly met only with infinite samples. For examination of wild-type layer 1 FSCs, we followed 92 cells, observing 20 exits from G1 and 16 exits from G2, suggesting a sampling of any specific point in the cell cycle about 18 times with an imaging window representing about (148 min/ 943 min) 16% of the average cell cycle. The document "Sampling Considerations for Live FUCCI Calculation Method" considers the general validity of the method we use when sampling is not necessarily evenly distributed around the cell cycle (because there are not infinite samples), and includes an estimate of a likely sampling bias and standard error for the data we used. For layer 1 FSCs, the estimated sampling bias was small (0 to 7.2%, mean 3.9% of the estimated cell cycle phase length) and the estimated standard error ranged from 18% to 30% (mean 24%) of the estimated cell cycle phase length for G1 and G2 of wild-type samples and those overexpressing CycE or JAK.

We are not aware of widespread use of imaging limited portions of the cell cycle to estimate the length of different phases or of the whole cell cycle. This approach is the only option available if it is not feasible to extend live imaging to exceed the cell cycle length of most of the cells being observed (generally, more than 24h). Additionally, when long-term imaging is possible it may still be better to choose short-term imaging. The imaging process can certainly impact normal physiology if samples must be put in new surroundings (as in our live imaging method) and perhaps also for *in situ* methods because of necessary surgery, behavioral limitations or illumination damage. Using only short imaging periods has the advantages that the signaling environment and normal behavior more likely remains intact. Those advantages may more than offset the systematic modest deficits in estimating mean cell cycle parameters due to heterogeneity within the population of cells examined. Each approach will be similarly limited by sampling statistics, according to the total observation time as a fraction of cell cycle duration. Using short imaging periods cannot, of course, explicitly describe cell by cell variations, which can be observed by imaging whole cell cycles. Short-period imaging may therefore find the greatest beneficial use when the key objective is to estimate mean cell cycle phase lengths in tightly-defined cell populations, as exemplified here for layer 1 FSCs and layer 2 FSCs.

Sampling Considerations for Live FUCCI calculation method

1 A model for random sampling of cell cycle

In this section we describe a stochastic model for random sampling of a two-stage cell cycle. The cell cycle is represented as the circumference of a circle of length z divided into two phases, a first ("green") of length y, a second ("red") of length $z - y$. By symmetry, we may assume that the first phase starts at angle 0. We mimic the experiment by assuming a viewing window of length x whose starting angle is θ (reading in an anti-clockwise direction). We assume θ has a uniform distribution on $(0, 2\pi)$. See Figure 1. The data come from properties of the lengths of the red and green segments that are viewed in the interval of length x.

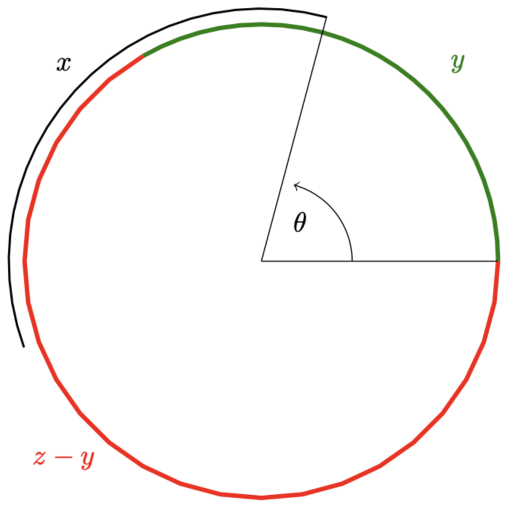

Figure 1: The green phase of length y shown on a circle of circumference z, the length of the cell cycle. The viewing window is of length x, and starts at an angle θ uniformly distributed over $(0, 2\pi)$.

1.1 Analysis when x, y, z are fixed

First we calculate the probability of several events related to this experiment, such as

- $\mathbb{P}(\mathrm{G})$, the probability that the entire observed region is green
- $\mathbb{P}(\mathrm{RG})$, the probability that the observation starts in the green region, and ends in the red region
- $\mathbb{P}(\mathrm{GR})$, the probability that the observation starts in the red region, and ends in the green region
- $\mathbb{P}(\mathrm{RGR})$, the probability that the observation starts in the red phase, covers the green region and ends in the green region
- $\mathbb{P}(\mathrm{R})$, the probability that the entire observed region is red
- $\mathbb{P}(\mathrm{G} \to \mathrm{R})$, the probability that an observation has green followed by red

and we also calculate $\mathbb{E}(G)$, the expected length of the observed green region.

There are two cases to consider: When $x < y$, elementary probabilistic arguments establish that

$$\begin{aligned} \mathbb{P}(\mathrm{G}) &= (y-x)/z, \quad \mathbb{P}(\mathrm{RG}) = x/z, \quad \mathbb{P}(\mathrm{RGR}) = 0, \quad \mathbb{P}(\mathrm{GR}) = x/z \\ \mathbb{P}(\mathrm{R}) &= (z-x-y)/z \text{ if } x+y<z; \quad 0 \text{ otherwise,} \end{aligned}$$

while if $y < x$, then

$$\begin{aligned} \mathbb{P}(\mathrm{G}) &= 0, \quad \mathbb{P}(\mathrm{RG}) = y/z, \quad \mathbb{P}(\mathrm{RGR}) = (x-y)/z, \quad \mathbb{P}(\mathrm{GR}) = y/z \\ \mathbb{P}(\mathrm{R}) &= (z-x-y)/z \text{ if } x+y<z; \quad 0 \text{ otherwise.} \end{aligned}$$

It follows that, in either case,

$$\mathbb{P}(\mathrm{G} \to \mathrm{R}) = \mathbb{P}(\mathrm{GR}) + \mathbb{P}(\mathrm{RGR}) = \frac{x}{z} \tag{1}$$

and

$$\mathbb{E}(G) = \frac{xy}{z}. \tag{2}$$

1.2 Estimating y

We will use equations (1) and (2) to estimate y from the data. To do this, begin by assuming that the values of y and z are fixed across n replicates of a given experimental condition, and write g_i for the length of the green segment observed in experiment i, and set $s_i = 1$ if experiment i results in an observation that has a green region followed by a red region, and 0 otherwise. From (2), we see that if x_i is the window length for experiment i, then $\mathbb{E}(g_i) = x_i y/z$, and so

$$\mathbb{E}\bar{g} := \mathbb{E}\left(\frac{1}{n}\sum_{i=1}^{n} g_i\right) = \left(\frac{1}{n}\sum_{i=1}^{n} x_i\right)\frac{y}{z} := \bar{x}\frac{y}{z}. \tag{3}$$

Similarly, from (1) we get

$$\mathbb{E}\bar{s} := \mathbb{E}\left(\frac{1}{n}\sum_{i=1}^{n} s_i\right) = \left(\frac{1}{n}\sum_{i=1}^{n} x_i\right)\frac{1}{z} = \bar{x}\frac{1}{z}. \tag{4}$$

The results in (3) and (4) suggest the ratio estimator $\hat{y}$ of y given by

$$\hat{y} = \frac{\bar{g}}{\bar{s}}, \tag{5}$$

since $y = \mathbb{E}\bar{g}/\mathbb{E}\bar{s}$.

Ratio estimators are biased, since (typically)

$$\mathbb{E}\hat{y} = \mathbb{E}\frac{\bar{g}}{\bar{s}} \neq \frac{\mathbb{E}\bar{g}}{\mathbb{E}\bar{s}} = y.$$

In order to estimate the bias in $\hat{y}$ we used the jackknife (see, for example, Choquet et al., 1999), which also provides an estimate of the variance of $\hat{y}$. The R package `bootstrap` was used for the analysis:

```
library(bootstrap)
# xdata is an n-row two-column matrix containing the values of
# (g_i, s_i) for given  experimental condition with $n$ observations
boottest <- function(xdata){
  n <- length(xdata[,1])
  thetaest <- mean(xdata[,1])/mean(xdata[,2])
  theta <- function(x,xdata){mean(xdata[x,1])/mean(xdata[x,2])}
  results <- jackknife(1:n,theta,xdata)
  return(c(thetaest,results))
```

The results are summarized in Table 1. Note that the corrected estimates are smaller than the original estimates, suggesting that the ratio estimator is biased upwards. The estimated standard error of the ratio estimator allows an assessment of the information carried about the average length of the green region in each experimental condition.

Dataset	Experiment	n	$\hat{y}$	bias	$\hat{y}_{\mathrm{corr}}$	SE
1*	WT layer 1 G2	68	527.00	28.33	498.67	130.58
2*	WT layer 1 G1	24	81.05	1.62	79.43	19.47
3	WT layer 2 G2	59	1957.50	606.79	1350.71	1251.45
4	WT layer 2 G1	11	407.00	160.76	246.24	312.79
5*	CycE layer 1 G2	31	390.73	27.84	362.89	118.01
6*	CycE layer 1 G1	14	23.64	0.00	23.64	4.27
7	CycE layer 2 G2	21	1005.00	461.75	543.25	847.21
8	CycE layer 2 G1	9	180.20	36.93	143.27	118.53
9*	JAK layer 1 G2	44	277.33	11.55	265.78	65.85
10*	JAK layer 1 G1	45	204.95	7.38	197.57	46.535
11	JAK layer 2 G2	32	701.16	121.22	579.94	327.90
12	JAK layer 2 G1	19	550.75	145.42	405.33	325.95

Table 1: n denotes sample size. SE denotes standard error, corr denotes corrected estimate. Entries marked with * indicate plausible error estimate from SE.

1.3 Accounting for randomness in y and z

Next, we need to consider the effects of randomness in the values of y and z from experiment to experiment. We begin with a more detailed look at $\mathbb{E}\hat{y}$, by considering (y, z) as an observation from a random variable (Y, Z); we assume the replicates $(Y_i, Z_i), i = 1, 2, \ldots, n$ are independent across n replicates from the same experimental condition. Averaging over the distribution of (Y, Z),

we find from (1) and (2) that

$$\mathbb{E}(G) = x\,\mathbb{E}\left(\frac{Y}{Z}\right)$$

and

$$\mathbb{P}(\mathrm{G} \to \mathrm{R}) = x\,\mathbb{E}\left(\frac{1}{Z}\right),$$

so that

$$\mathbb{E}\bar{g} = \bar{x}\,\mathbb{E}\left(\frac{Y}{Z}\right), \quad \mathbb{E}\bar{s} = \bar{x}\,\mathbb{E}\left(\frac{1}{Z}\right). \tag{6}$$

and

$$\frac{\mathbb{E}\bar{g}}{\mathbb{E}\bar{s}} = \mathbb{E}\left(\frac{Y}{Z}\right) / \,\mathbb{E}\left(\frac{1}{Z}\right). \tag{7}$$

To relate the ratio on the right of (7) to the parameter $\mathbb{E}Y$ we are trying to estimate, we need to make some assumptions. For example, if the distribution of Y is closely concentrated around its mean (so Y is almost constant), we might approximate $\mathbb{E}(Y/Z)$ by $\mathbb{E}(Y)\mathbb{E}(1/Z)$, so that the ratio estimator in (5) is appropriate.

An alternative is to use an explicit model for (Y, Z), one of which we describe in the next section.

1.4 A model for (Y, Z)

One explicit model takes $Y = W_1, Z = W_1 + W_2$, where W_i has a gamma distribution with shape parameter $r_i > 0$ and scale parameter $1/\lambda > 0$, and W_1 and W_2 are independent. The density of W_i is

$$f_i(x) = \frac{\lambda^{r_i} x^{r_i - 1} e^{-\lambda x}}{\Gamma(r_i)}, \quad x > 0,$$

illustrated in Figure 2.

We have

$$\mathbb{E}Y = \frac{r_1}{\lambda}, \quad \mathrm{Var}Y = \frac{r_1}{\lambda^2}.$$

It follows that Z has a gamma distribution with parameters $r_1 + r_2$ and λ, and furthermore Y/Z and Z are independent. As a consequence,

$$\mathbb{E}Y = \mathbb{E}\left(\frac{Y}{Z}\,Z\right) = \mathbb{E}\left(\frac{Y}{Z}\right)\mathbb{E}Z,$$

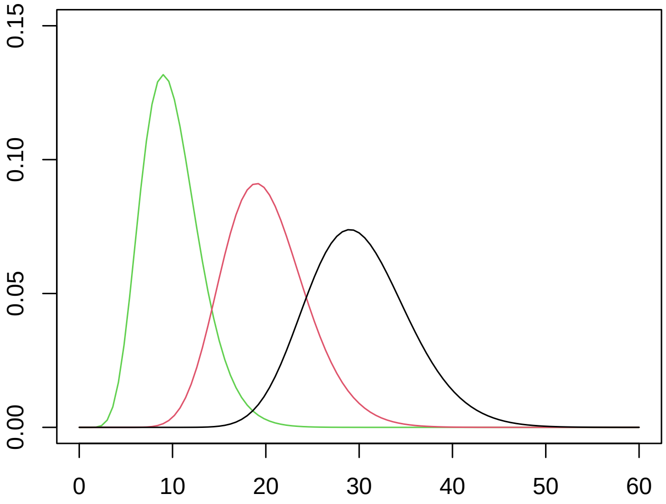

Figure 2: Illustrative densities of the green region W_1 (green line, shape =1, mean = 10), the red region W_2 (red line, shape = 1, mean = 20), $W_1 + W_2$ (black line, shape = 1, mean = 30).

so that

$$\mathbb{E}\left(\frac{Y}{Z}\right) = \frac{\mathbb{E}Y}{\mathbb{E}Z} = \frac{r_1}{r_1 + r_2}.$$

We also have

$$\mathbb{E}\left(\frac{1}{Z}\right) = \frac{\lambda}{r_1 + r_2 - 1}$$

The upshot of these results is that (7) becomes

$$\frac{\mathbb{E}\bar{g}}{\mathbb{E}\bar{s}} = \frac{r_1}{\lambda}\frac{r_1 + r_2 - 1}{r_1 + r_2} \approx \frac{r_1}{\lambda} = \mathbb{E}Y,$$

if for example $r_1 + r_2$ is large. It follows that the ratio estimator in (5) is still appropriate.

References

Choquet D, L'Ecuyer P, Léger C. Bootstrap confidence intervals for ratios of expectations. *ACM Transactions on Modeling and Computer Simulation*, **9**, 326–348, 1999.
